# Accurate multiple sequence-structure alignment of RNA sequences using combinatorial optimization

**DOI:** 10.1186/1471-2105-8-271

**Published:** 2007-07-27

**Authors:** Markus Bauer, Gunnar W Klau, Knut Reinert

**Affiliations:** 1 Department of Mathematics and Computer Science, Free University Berlin, 14195 Berlin, Germany; 2 International Max Planck Research School for Computational Biology and Scientific Computing, Berlin, Germany; 3 DFG Research Center Matheon, Berlin, Germany

## Abstract

**Background:**

The discovery of functional non-coding RNA sequences has led to an increasing interest in algorithms related to RNA analysis. Traditional sequence alignment algorithms, however, fail at computing reliable alignments of low-homology RNA sequences. The spatial conformation of RNA sequences largely determines their function, and therefore RNA alignment algorithms have to take structural information into account.

**Results:**

We present a graph-based representation for sequence-structure alignments, which we model as an integer linear program (ILP). We sketch how we compute an optimal or near-optimal solution to the ILP using methods from combinatorial optimization, and present results on a recently published benchmark set for RNA alignments.

**Conclusion:**

The implementation of our algorithm yields better alignments in terms of two published scores than the other programs that we tested: This is especially the case with an increasing number of input sequences. Our program LARA is freely available for academic purposes from .

## 1 Background

In recent years, research in RNA sequences and structures has dramatically increased: the discovery of functionally important, not protein-coding, RNA sequences has challenged the traditional picture of the flow of genetic information from DNA via RNA to proteins as functional units. It is now well-established that RNA molecules introduce an additional layer in genetic information processing. They play a significant active role in cell and developmental biology and carry out many tasks that were previously attributed exclusively to proteins. One of the most eminent examples is the class of microRNAs [1,2], an abundant class of small functional RNAs that regulate gene expression by binding to a target in the mRNA. Other examples include snoRNAs, which modify ribosomal RNA [3], *signal recognition particle *RNAs [4], *cis*-acting regulatory elements, and piRNAs [5], a novel class of ncRNAs whose function is still unclear. It is likely that only a small fraction of regulatory RNAs has been identified so far and that many more have yet to be discovered [6].

Computational analyses have contributed largely to the discovery and advancement of biological knowledge. Heuristic methods, such as BLAST [7], or exact approaches based on dynamic programming, such as the Smith-Waterman algorithm [8], are used as everyday tools to analyze DNA and protein sequences. In case of RNA sequences, sequence information alone is not sufficient anymore. An RNA sequence folds back onto itself and forms hydrogen bonds between nucleotides. These bonds lead to the distinctive *secondary structure *of an RNA sequence.

**Figure 1:**
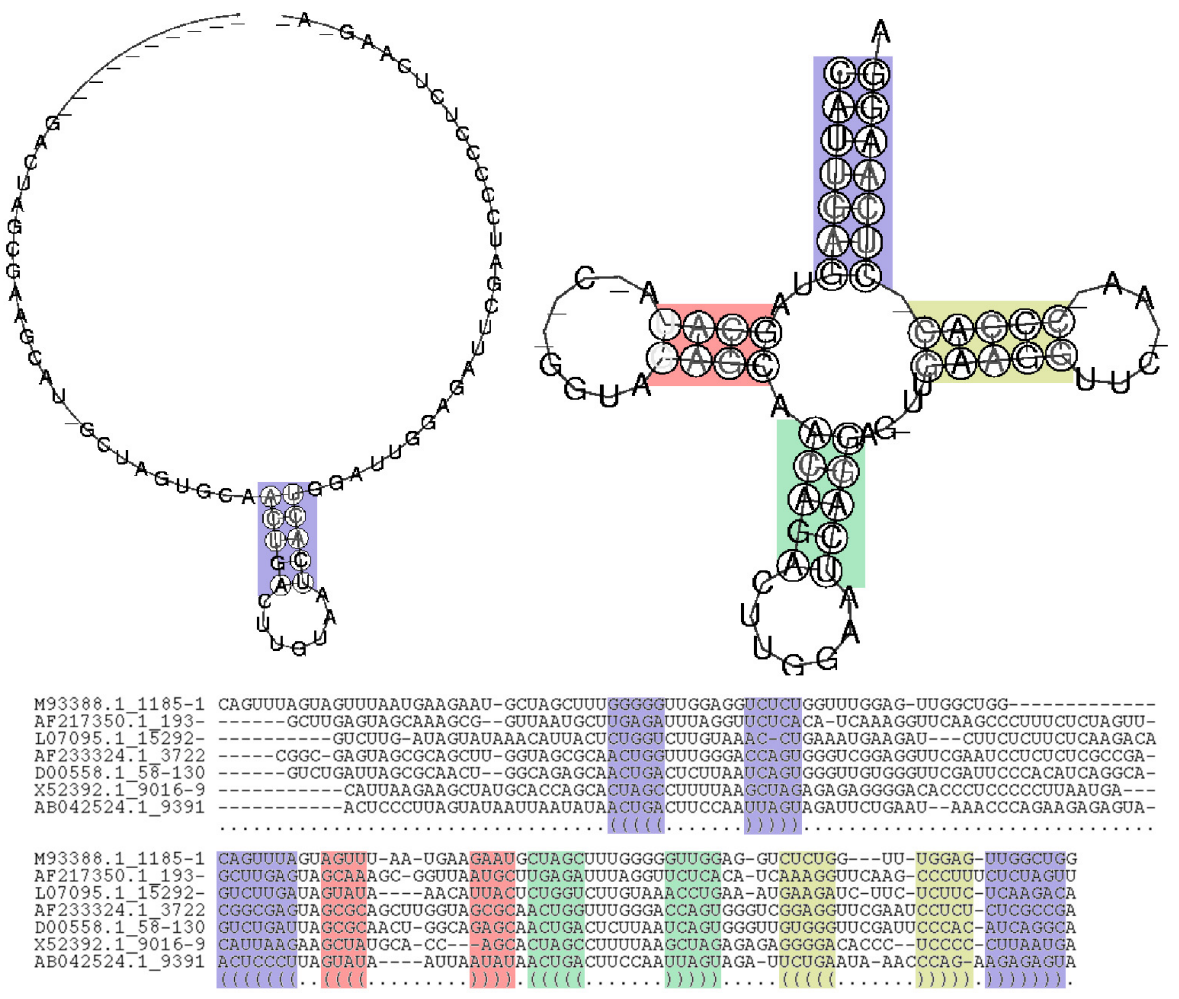
**Comparison between sequence and sequence-structure alignments**. Comparison between sequence-based (upper, computed by the CLUSTALW program [60]) and sequence-structure-based alignment (lower, computed by LARA, an implementation of our new approach). The left and right consensus structures are based on the ClustalW and LaRA alignment, respectively (consensus structures generated using RNAalifold [11]).

RNA sequences evolve more rapidly than the structure they are forming, because their evolutionary behavior follows the *structure-function *paradigm: RNA molecules with different sequences but same or similar secondary structure are likely to belong to the same functional family, in which the secondary structure is conserved by selective pressure. Hence, computational analysis of RNA molecules inevitably involves considering secondary structure information in addition to the primary sequence. Computing *sequence-structure alignments *is a key step in many important applications. These include finding homologous structures of known ncRNA families [9], phylogenetic fingerprinting (as conducted for example for the ITS2 database [10]), or the computation of a consensus structure of a set of RNA molecules [11]. A recent study shows that pure sequence-based pairwise alignments are unable to produce satisfactory results if the pairwise sequence identity drops below 50 to 60% [12]. Figure 1 illustrates this situation and shows two different alignments of seven tRNA sequences with a pairwise sequence identity of 39%, where the upper alignment is based on sequence information alone and the lower alignment additionally rewards the conservation of structural elements. One can clearly see that the sequence-based alignment is unable to preserve the typical tRNA-cloverleaf structure, whereas the structural alignment conserves the structural features of the input sequences.

Unfortunately, considering structural information adds an additional level of complexity to the problem of aligning two or several sequences. In the remainder of this section, we present a classification of structural alignment problem variants including previous work. Section 2 describes our new approach to multiple sequence-structure alignment. We employ methods from mathematical programming and solve the problem as an integer linear program resulting from a graph-theoretical reformulation. Section 3 is dedicated to an extensive computational study. We describe LARA, the freely available implementation of our novel approach, and present detailed results of a comparative study including state-of-the-art programs on a recently published benchmark database of structural alignments. The results show that on average our software is currently the best program in terms of alignment quality, outperforming other programs with an increasing number of input sequences. Finally, we discuss our results and suggest future research directions in Sect. 4.

In contrast to previous work [13-15] this article describes a full integer linear programming (ILP) formulation that does include arbitrary gap costs and an extensive performance analysis of our implementation for the first time. Due to page limits the mathematical fundament and all proofs are omitted: the interested reader is referred to the companion paper [16] that focuses on an in-depth description of the mathematical properties of the intricate multiple case containing all proofs.

### 1.1 Previous approaches

**Figure 2:**
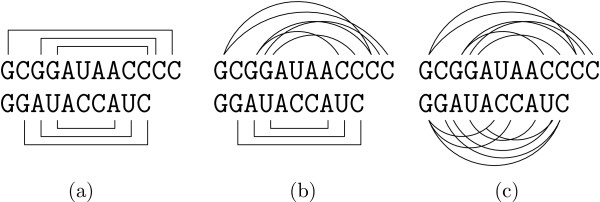
**Input scenarios**. Different input alignment scenarios of RNA sequences (pairwise case): (a) the alignment of two known structures, (b) of one known and one unknown structure, and (c) of two unknown structures.

Depending on the available knowledge about the (putative) structures that we want to align, there are three different alignment scenarios for two RNA structures, which readily extend to the multiple case. Figure 2 gives a cartoon illustration of the three scenarios.

1. *Structure-to-structure *alignments align two known secondary structures, typically the *minimum free energy *structures. This scenario applies if one searches for common structural motifs that are shared by both structures and there is reason to believe that the secondary structures are correct.

2. *Structure-to-unknown *alignments align a given structure to a sequence with unknown structure. Applications are finding homologous sequences by inferring a consensus structure to a sequence (this is done, for example, in the verification phase of the FASTR package [9]), or finding new family members of ncRNA families: This problem has recently sparked considerable interest in the context of searching homologous structures of noncoding-RNAs in large genomic sequences. See [17] for a survey.

3. In the *unknown-to-unknown *alignment problem, no previous structural information is given. It applies when two RNA sequences are suspected to share a common, but still unknown, structure. We constrain the space of possible structures by the entire set of possible Watson-Crick and wobble pairs. A reduction of the size of this space is possible, for instance, by applying a folding algorithm to obtain the base pair probabilities [18] and then considering only those interactions whose probabilities are above a certain threshold.

**Figure 3:**
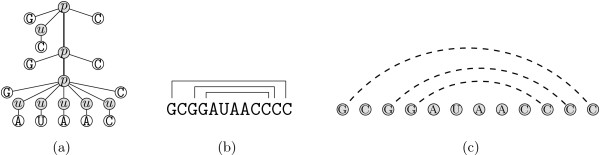
**RNA representations**. Different models representing RNA structures: (a) tree representation, (b) annotated sequences, and (c) graph-based models.

There are four major alignment models for RNA structures that tackle the previous described alignment scenarios: *annotated sequences*, *tree models*, *probabilistic models*, and *graph-based models*. We give small examples for each model in Fig. 3: Note that we did not show an example of probabilistic models because the representation of probabilistic and tree models are the same. The underlying algorithms, however, are completely different. Table 1 classifies previous work in the area of structural RNA alignment according to the different models and scenarios.

**Table 1:** Classification of previous work

	tree-based	annotated sequences	probabilistic	graph-based
structure-to-structure	[20-22,61]	[22,25,26,62]	[34]	[14,41,43]
structure-to-unknown	--	[22]	[34,38]	[14,41,43]
unknown-to-unknown	--	[22,27–29,31]	[37]	[14,41,43]

#### Tree-based models

Tree-based structural alignment algorithms view an RNA secondary structure as a tree. Depending on the particular model (either tree-editing [19] or tree alignment [20]), one either searches for the minimal number of operations (node inserting, node deletion, and node substitution) to transform one tree into the other, or into a common supertree. Algorithms employing the model from [20] have time complexities in *O*(*n*^4^), thus making the computation expensive. Here and in the following, *n *denotes the size of the longest sequence. Tree-alignment algorithms have complexities that are on average only slightly worse than conventional sequence alignment. More precisely, their running time is in *O*(*n*^2^·Δ^2^), where Δ denotes the maximum number of branches of a multiloop in the input structures.

A tool that builds upon the tree paradigm is RNAFORESTER [21]. It computes multiple structure-to-structure alignments of RNA sequences by performing tree-alignment in a progressive fashion.

#### Annotated sequences

We call a sequence that is augmented by structural information an *annotated sequence*. Classical dynamic programming (DP) algorithms can be extended to annotated sequences. The DP solution for the structure-to-structure and structure-to-unknown problem then typically requires *O*(*n*^4^) and *O*(*n*^3^) in time and space, respectively. Bafna, Muthukrishnan, and Ravi describe an algorithm that simultaneously aligns the sequence and secondary structure of two RNA sequences [22]. Their method runs in time *O*(*n*^4^), which still does not make it applicable to instances of realistic size. Eddy [23] proposes an algorithm that reduces the memory consumption to *O*(*n*^2 ^log *n*). The STRAL tool [24] uses the values of the *base pair probability matrices*, as given by the partition function [18], to compute the maximal pairing probability of a single nucleotide and to align the sequences in a CLUSTALW-like fashion.

In the restricted structure-to-structure scenario, one can resort to more sophisticated edit-models like the one proposed by Jiang in [25] where the authors specify operations both on the sequence and the structure level. The dynamic programming algorithm is in *O*(*n*^4^), making the computation rather tedious for longer sequences. A program that implements the Jiang model is MARNA [26]: it computes pairwise sequence-structure alignments, but is additionally able to compute multiple alignments. To this end, MARNA computes all pairwise structural alignment and uses T-COFFEE to compute the actual multiple alignment incorporating the structural information of the pairwise alignments.

The unknown-to-unknown scenario requires the simultaneous computation of the alignment and consensus structure. The computational problem of simultaneously considering sequence and structure of an RNA molecule was initially addressed by Sankoff in [27], where the author proposed a DP algorithm to align and fold a set of RNA sequences at the same time. The CPU and memory requirements of the original algorithm are *O*(*n*^3*k*^) and *O*(*n*^2*k*^), respectively, where *k *is the number of sequences and *n *is their maximal length. Current implementations modify Sankoff's algorithm by imposing limits on the size or shape of substructures, e.g., DYNALIGN [28,29], or FOLDALIGN [30] that combine a sliding window and banded alignment approach. Hofacker, Bernhart, and Stadler [31] have presented the PMMULTI software to align base pair probability matrices. Their recursions are essentially the same as the ones given by Sankoff in [27] and subsequently used for sequence-structure alignment by Bafna et al. in [22] with the only difference that they consider probabilities instead of fixed structures. By banding the range of possible alignment positions they bring the time and space complexity of the pairwise case down to *O*(*n*^4^) and *O*(*n*^3^), respectively. For the multiple case, they align consensus base pair probability matrices in a progressive fashion. Similar in spirit are FOLDALIGNM [32] or LOCARNA [33], two recent reimplementations of the PMMULTI approach. FOLDALIGNM provides both several restrictions on the alignment and a two-stage procedure to fill the DP matrix: this further reduces the running time to *O*(*n*^2^*δ*^2^) where *n *is the length of the longer sequence and *d *is the maximal length difference of the alignment of two subsequences. LOCARNA on the other hand takes advantage of the sparse base pair probabilities matrices to reduce the running time.

#### Probabilistic models

Eddy and Durbin [34] describe *covariance models *for measuring the secondary structure and primary sequence consensus of RNA sequence families. They present algorithms for analyzing and comparing RNA sequences as well as database search techniques. Since the basic operation in their approach is an expensive dynamic programming algorithm, their algorithms cannot analyze sequences longer than 150–200 nucleotides. Therefore, recent approaches reduce the running time by incorporating additional information, e.g. Holmes et al.'s STEMLOC [35,36] where the authors propose the concept of *alignment/fold envelopes *that constrain possible alignments. Along these lines, in [37] the authors keep a set of probabilistically derived alignment positions fixed: these alignment positions serve subsequently as anchors for the structural alignment which prune away large parts of the search space. The authors of [38] describe a method based on conditional random fields to align an RNA sequence with known structure to one with unknown structure. They estimate their parameters using conditional random fields and compute the alignment using the recursions from [39].

#### Graph-based models

Kececioglu [40] has introduced a graph-theoretical model for the classical primary sequence alignment problem. In [41] the authors present a first extension of this model to RNA structures and propose a branch-and-cut approach based on an integer linear programming formulation. Based on this formulation and inspired by the successful application of Lagrangian relaxation by Lancia and Caprara [42] to the related *contact map overlap problem*, in [43] the authors switch from branch-and-cut to the Lagrangian relaxation technique. They are able to solve instances a magnitude larger by simultaneously reducing the running time significantly. In [44] the authors give a graph-theoretic model for the computation of multiple sequence alignments with arbitrary gap costs. In the next section we will combine the formulations given in [43] and [44], resulting in a novel graph-based formulation for sequence-structure alignment with arbitrary gap costs.

Note that the graph-based model naturally deals with all three alignment scenarios. In addition, unlike other algorithmic approaches, the graph-based algorithms do not restrict the input in any way and hence can handle arbitrary *pseudoknots*: Pseudoknots have been shown to play important roles in a variety of biological processes, see [45] for a recent review. Most DP-based algorithms assume nested secondary structures to compute subproblems efficiently. Few exceptions exist, for example [46], but these algorithms are always restricted to certain classes of pseudoknots (like H-type pseudoknots) and do not handle the general case.

## 2 Results

This section deals with our novel graph-based approach to structural RNA alignment. We first give the problem definition and then describe the graph-theoretical model we use, which combines the models presented in [43] and [44]. We convert the nucleotides of the input sequences into vertices of a graph, and we add edges between the vertices that represent either structural information or possible alignments of pairs of nucleotides. Based on the graph model we develop an integer linear programming formulation. We find solutions using an algorithmic approach employing methods from combinatorial optimization. For sake of simplicity, we will limit the description to the two-sequence case. We want to stress, however, that the model can be extended to the multiple case without changing the core algorithms and ideas. The interested reader is referred to an extensive theoretical description including proofs and a computational complexity discussion appearing elsewhere [16].

### 2.1 Graph-theoretical model for structural RNA alignment

#### Problem definition

Given two RNA sequences, we denote by A
 MathType@MTEF@5@5@+=feaafiart1ev1aaatCvAUfKttLearuWrP9MDH5MBPbIqV92AaeXatLxBI9gBaebbnrfifHhDYfgasaacH8akY=wiFfYdH8Gipec8Eeeu0xXdbba9frFj0=OqFfea0dXdd9vqai=hGuQ8kuc9pgc9s8qqaq=dirpe0xb9q8qiLsFr0=vr0=vr0dc8meaabaqaciaacaGaaeqabaqabeGadaaakeaat0uy0HwzTfgDPnwy1egaryqtHrhAL1wy0L2yHvdaiqaacqWFaeFqaaa@3820@ an alignment of the two sequences. Let *s*_*S*_(A
 MathType@MTEF@5@5@+=feaafiart1ev1aaatCvAUfKttLearuWrP9MDH5MBPbIqV92AaeXatLxBI9gBaebbnrfifHhDYfgasaacH8akY=wiFfYdH8Gipec8Eeeu0xXdbba9frFj0=OqFfea0dXdd9vqai=hGuQ8kuc9pgc9s8qqaq=dirpe0xb9q8qiLsFr0=vr0=vr0dc8meaabaqaciaacaGaaeqabaqabeGadaaakeaat0uy0HwzTfgDPnwy1egaryqtHrhAL1wy0L2yHvdaiqaacqWFaeFqaaa@3820@) be the sequence score of alignment A
 MathType@MTEF@5@5@+=feaafiart1ev1aaatCvAUfKttLearuWrP9MDH5MBPbIqV92AaeXatLxBI9gBaebbnrfifHhDYfgasaacH8akY=wiFfYdH8Gipec8Eeeu0xXdbba9frFj0=OqFfea0dXdd9vqai=hGuQ8kuc9pgc9s8qqaq=dirpe0xb9q8qiLsFr0=vr0=vr0dc8meaabaqaciaacaGaaeqabaqabeGadaaakeaat0uy0HwzTfgDPnwy1egaryqtHrhAL1wy0L2yHvdaiqaacqWFaeFqaaa@3820@ including gap penalties, and let *s*_*P*_(A
 MathType@MTEF@5@5@+=feaafiart1ev1aaatCvAUfKttLearuWrP9MDH5MBPbIqV92AaeXatLxBI9gBaebbnrfifHhDYfgasaacH8akY=wiFfYdH8Gipec8Eeeu0xXdbba9frFj0=OqFfea0dXdd9vqai=hGuQ8kuc9pgc9s8qqaq=dirpe0xb9q8qiLsFr0=vr0=vr0dc8meaabaqaciaacaGaaeqabaqabeGadaaakeaat0uy0HwzTfgDPnwy1egaryqtHrhAL1wy0L2yHvdaiqaacqWFaeFqaaa@3820@) be the score of structural features that are conserved by the alignment A
 MathType@MTEF@5@5@+=feaafiart1ev1aaatCvAUfKttLearuWrP9MDH5MBPbIqV92AaeXatLxBI9gBaebbnrfifHhDYfgasaacH8akY=wiFfYdH8Gipec8Eeeu0xXdbba9frFj0=OqFfea0dXdd9vqai=hGuQ8kuc9pgc9s8qqaq=dirpe0xb9q8qiLsFr0=vr0=vr0dc8meaabaqaciaacaGaaeqabaqabeGadaaakeaat0uy0HwzTfgDPnwy1egaryqtHrhAL1wy0L2yHvdaiqaacqWFaeFqaaa@3820@. We now aim at maximizing the combined sequence-structure score, that is, we search for an alignment A∗
 MathType@MTEF@5@5@+=feaafiart1ev1aaatCvAUfKttLearuWrP9MDH5MBPbIqV92AaeXatLxBI9gBaebbnrfifHhDYfgasaacH8akY=wiFfYdH8Gipec8Eeeu0xXdbba9frFj0=OqFfea0dXdd9vqai=hGuQ8kuc9pgc9s8qqaq=dirpe0xb9q8qiLsFr0=vr0=vr0dc8meaabaqaciaacaGaaeqabaqabeGadaaakeaat0uy0HwzTfgDPnwy1egaryqtHrhAL1wy0L2yHvdaiqaacqWFaeFqdaahaaWcbeqaaiabgEHiQaaaaaa@393C@ with

sS(A∗)+sP(A∗)=max⁡AsS(A)+sP(A).
 MathType@MTEF@5@5@+=feaafiart1ev1aaatCvAUfKttLearuWrP9MDH5MBPbIqV92AaeXatLxBI9gBaebbnrfifHhDYfgasaacH8akY=wiFfYdH8Gipec8Eeeu0xXdbba9frFj0=OqFfea0dXdd9vqai=hGuQ8kuc9pgc9s8qqaq=dirpe0xb9q8qiLsFr0=vr0=vr0dc8meaabaqaciaacaGaaeqabaqabeGadaaakeaacqWGZbWCdaWgaaWcbaGaem4uamfabeaakiadmcOGOaakt0uy0HwzTfgDPnwy1egaryqtHrhAL1wy0L2yHvdaiqaacqWFaeFqdGaJaYbaaSqajWiGbGaJakadmcOHxiIkaaGccWaJakykaKIaey4kaSIaem4Cam3aaSbaaSqaaiabdcfaqbqabaGccqGGOaakcqWFaeFqdaahaaWcbeqaaiabgEHiQaaakiabcMcaPiabg2da9maaxababaGagiyBa0MaeiyyaeMaeiiEaGhaleaacqWFaeFqaeqaaOGaem4Cam3aaSbaaSqaaiabdofatbqabaGccqGGOaakcqWFaeFqcqGGPaqkcqGHRaWkcqWGZbWCdaWgaaWcbaGaemiuaafabeaakiabcIcaOiab=bq8bjabcMcaPiabc6caUaaa@61E3@

**Figure 4:**
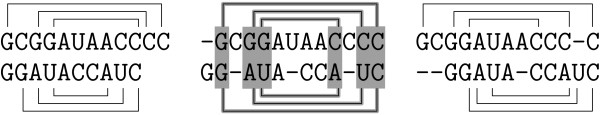
**Problem statement**. Given the annotated sequences on the left side as the input, we search for an alignment maximizing the sequence plus the induced structure score. The alignment in the middle conserves the entire annotation (highlighted in grey), whereas the alignment on the right hand side maximizes the sequence score and does not conserve any structure.

Figure 4 gives a toy example showing two annotated sequences and two possible alignments, one maximizing the score of sequence and structure, and the other one just the sequence score alone. This problem definition comprises the one addressed by Bafna et al. in [22]: Our model, however, also allows tertiary elements, which is not covered by their recursions.

#### Basic model

Let *s *= *s*_1_, ..., *s*_*n *_be a sequence of length *n *over the alphabet Σ = {*A*, *C*, *G*, *U*}. A pair (*s*_*i*_*, s*_*j*_) is called an *interaction *if *i < j*, and nucleotide *i *pairs with *j*. In most cases, these pairs will be Watson-Crick or wobble base pairs. The set *p *of interactions is called the *annotation *of sequence *s*. Two interactions (*s*_*k*_*, s*_*l*_) and (*s*_*m*_*, s*_*o*_) are said to be *inconsistent*, if they share one base; they form a *pseudoknot *if they "cross" each other, that is, if *k *<*m *<*l *<*o *or *m *<*k *<*o *<*l*. A pair (*s*, *p*) is called an *annotated sequence*. Note that a structure where no pair of interactions is inconsistent with each other forms a valid secondary structure of an RNA sequence, possibly with pseudoknots.

**Figure 5:**
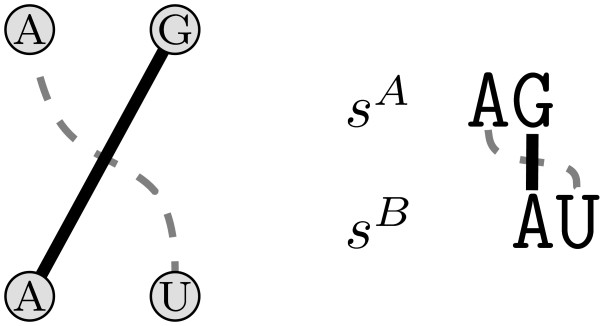
**Crossing lines**. Sequences *s*^*A *^= *AG *and *s*^*B *^= *AU *are given. The solid line between *G *and *A *represent the alignment of these two nucleotides. If we added the gray dashed line, this would induce an ordering conflict.

We are given two annotated sequences (*s*^*A*^, *p*^*A*^) and (*s*^*B*^, *p*^*B*^) and model the input as a *structural graph G*_*S *_= (*V, L*). The set *V *denotes the vertices of the graph, in this case the bases of the sequences, and we write viA
 MathType@MTEF@5@5@+=feaafiart1ev1aaatCvAUfKttLearuWrP9MDH5MBPbIqV92AaeXatLxBI9gBaebbnrfifHhDYfgasaacH8akY=wiFfYdH8Gipec8Eeeu0xXdbba9frFj0=OqFfea0dXdd9vqai=hGuQ8kuc9pgc9s8qqaq=dirpe0xb9q8qiLsFr0=vr0=vr0dc8meaabaqaciaacaGaaeqabaqabeGadaaakeaacqWG2bGDdaqhaaWcbaGaemyAaKgabaGaemyqaeeaaaaa@30B4@ and viB
 MathType@MTEF@5@5@+=feaafiart1ev1aaatCvAUfKttLearuWrP9MDH5MBPbIqV92AaeXatLxBI9gBaebbnrfifHhDYfgasaacH8akY=wiFfYdH8Gipec8Eeeu0xXdbba9frFj0=OqFfea0dXdd9vqai=hGuQ8kuc9pgc9s8qqaq=dirpe0xb9q8qiLsFr0=vr0=vr0dc8meaabaqaciaacaGaaeqabaqabeGadaaakeaacqWG2bGDdaqhaaWcbaGaemyAaKgabaGaemOqaieaaaaa@30B6@ for the *i*th base in sequence *A *and *B*, respectively. The set *L *contains undirected *alignment edges *between vertices of sequences *A *and *B*, for sake of better distinction called *lines*. A line *l *∈ *L *with *l *= (vkA,vlB
 MathType@MTEF@5@5@+=feaafiart1ev1aaatCvAUfKttLearuWrP9MDH5MBPbIqV92AaeXatLxBI9gBaebbnrfifHhDYfgasaacH8akY=wiFfYdH8Gipec8Eeeu0xXdbba9frFj0=OqFfea0dXdd9vqai=hGuQ8kuc9pgc9s8qqaq=dirpe0xb9q8qiLsFr0=vr0=vr0dc8meaabaqaciaacaGaaeqabaqabeGadaaakeaacqWG2bGDdaqhaaWcbaGaem4AaSgabaGaemyqaeeaaOGaeiilaWIaemODay3aa0baaSqaaiabdYgaSbqaaiabdkeacbaaaaa@35B2@) represents the alignment of the *k*-th character in sequence *A *with the *l*-th character in sequence *B*. By *s*(*l*) and *t*(*l*) we refer to the adjacent vertices of line *l *in sequence *A *and *B*, respectively. A subset ℒ
 MathType@MTEF@5@5@+=feaafiart1ev1aaatCvAUfKttLearuWrP9MDH5MBPbIqV92AaeXatLxBI9gBaebbnrfifHhDYfgasaacH8akY=wiFfYdH8Gipec8Eeeu0xXdbba9frFj0=OqFfea0dXdd9vqai=hGuQ8kuc9pgc9s8qqaq=dirpe0xb9q8qiLsFr0=vr0=vr0dc8meaabaqaciaacaGaaeqabaqabeGadaaakeaat0uy0HwzTfgDPnwy1egaryqtHrhAL1wy0L2yHvdaiqaacqWFsectaaa@376D@ ⊂ *L *represents a *valid sequence alignment *of sequence *A *and *B*, if there are no two lines *k*, *l *∈ ℒ
 MathType@MTEF@5@5@+=feaafiart1ev1aaatCvAUfKttLearuWrP9MDH5MBPbIqV92AaeXatLxBI9gBaebbnrfifHhDYfgasaacH8akY=wiFfYdH8Gipec8Eeeu0xXdbba9frFj0=OqFfea0dXdd9vqai=hGuQ8kuc9pgc9s8qqaq=dirpe0xb9q8qiLsFr0=vr0=vr0dc8meaabaqaciaacaGaaeqabaqabeGadaaakeaat0uy0HwzTfgDPnwy1egaryqtHrhAL1wy0L2yHvdaiqaacqWFsectaaa@376D@ such that *k *and *l *cross or touch each other [40]. Crossing or touching lines induce ordering conflicts in the alignment (see Fig. 5 for an illustration). We denote with the set CL
 MathType@MTEF@5@5@+=feaafiart1ev1aaatCvAUfKttLearuWrP9MDH5MBPbIqV92AaeXatLxBI9gBaebbnrfifHhDYfgasaacH8akY=wiFfYdH8Gipec8Eeeu0xXdbba9frFj0=OqFfea0dXdd9vqai=hGuQ8kuc9pgc9s8qqaq=dirpe0xb9q8qiLsFr0=vr0=vr0dc8meaabaqaciaacaGaaeqabaqabeGadaaakeaat0uy0HwzTfgDPnwy1egaryqtHrhAL1wy0L2yHvdaiqaacqWFce=qdaWgaaWcbaGaemitaWeabeaaaaa@3971@ the collection of all maximal sets of mutually conflicting lines.

**Figure 6:**
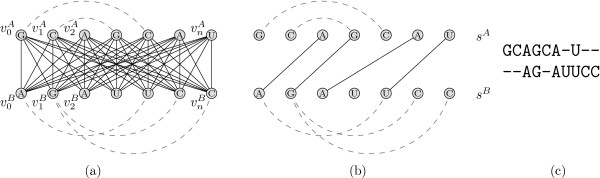
**Initial graph model**. (a) Initial graph model representing two annotated sequences *s*^*A *^= GCAGCAU and *s*^*B *^= AGAUUCC. Solid lines represent lines, dashed lines represent interaction edges. Please note that in this toy example minimum loop lengths constraints on the interaction edges are violated for sake of compactness of the illustration. Interactions (v1B,vn−1B
 MathType@MTEF@5@5@+=feaafiart1ev1aaatCvAUfKttLearuWrP9MDH5MBPbIqV92AaeXatLxBI9gBaebbnrfifHhDYfgasaacH8akY=wiFfYdH8Gipec8Eeeu0xXdbba9frFj0=OqFfea0dXdd9vqai=hGuQ8kuc9pgc9s8qqaq=dirpe0xb9q8qiLsFr0=vr0=vr0dc8meaabaqaciaacaGaaeqabaqabeGadaaakeaacqWG2bGDdaqhaaWcbaGaeGymaedabaGaemOqaieaaOGaeiilaWIaemODay3aa0baaSqaaiabd6gaUjabgkHiTiabigdaXaqaaiabdkeacbaaaaa@3726@) and (v1B,vnB
 MathType@MTEF@5@5@+=feaafiart1ev1aaatCvAUfKttLearuWrP9MDH5MBPbIqV92AaeXatLxBI9gBaebbnrfifHhDYfgasaacH8akY=wiFfYdH8Gipec8Eeeu0xXdbba9frFj0=OqFfea0dXdd9vqai=hGuQ8kuc9pgc9s8qqaq=dirpe0xb9q8qiLsFr0=vr0=vr0dc8meaabaqaciaacaGaaeqabaqabeGadaaakeaacqWG2bGDdaqhaaWcbaGaeGymaedabaGaemOqaieaaOGaeiilaWIaemODay3aa0baaSqaaiabd6gaUbqaaiabdkeacbaaaaa@3549@) are in conflict with each other, (v0B,vn−2B
 MathType@MTEF@5@5@+=feaafiart1ev1aaatCvAUfKttLearuWrP9MDH5MBPbIqV92AaeXatLxBI9gBaebbnrfifHhDYfgasaacH8akY=wiFfYdH8Gipec8Eeeu0xXdbba9frFj0=OqFfea0dXdd9vqai=hGuQ8kuc9pgc9s8qqaq=dirpe0xb9q8qiLsFr0=vr0=vr0dc8meaabaqaciaacaGaaeqabaqabeGadaaakeaacqWG2bGDdaqhaaWcbaGaeGimaadabaGaemOqaieaaOGaeiilaWIaemODay3aa0baaSqaaiabd6gaUjabgkHiTiabikdaYaqaaiabdkeacbaaaaa@3726@) and (v1B,vnB
 MathType@MTEF@5@5@+=feaafiart1ev1aaatCvAUfKttLearuWrP9MDH5MBPbIqV92AaeXatLxBI9gBaebbnrfifHhDYfgasaacH8akY=wiFfYdH8Gipec8Eeeu0xXdbba9frFj0=OqFfea0dXdd9vqai=hGuQ8kuc9pgc9s8qqaq=dirpe0xb9q8qiLsFr0=vr0=vr0dc8meaabaqaciaacaGaaeqabaqabeGadaaakeaacqWG2bGDdaqhaaWcbaGaeGymaedabaGaemOqaieaaOGaeiilaWIaemODay3aa0baaSqaaiabd6gaUbqaaiabdkeacbaaaaa@3549@) form a pseudoknot. Sequence *s*^*A *^contains only nested interactions. (b) A subset of all possible lines is shown representing the alignment (c).

We extend the original graph *G*_*S *_= (*V*, *L*) by the edge set *I *to model the annotation of the input sequences in our graph. Consequently, we have *interaction edges *between vertices of the same sequence, i.e., an edge (viA,vjA
 MathType@MTEF@5@5@+=feaafiart1ev1aaatCvAUfKttLearuWrP9MDH5MBPbIqV92AaeXatLxBI9gBaebbnrfifHhDYfgasaacH8akY=wiFfYdH8Gipec8Eeeu0xXdbba9frFj0=OqFfea0dXdd9vqai=hGuQ8kuc9pgc9s8qqaq=dirpe0xb9q8qiLsFr0=vr0=vr0dc8meaabaqaciaacaGaaeqabaqabeGadaaakeaacqWG2bGDdaqhaaWcbaGaemyAaKgabaGaemyqaeeaaOGaeiilaWIaemODay3aa0baaSqaaiabdQgaQbqaaiabdgeabbaaaaa@35A8@) representing the interaction between nucleotides *i *and *j *in sequence *A*. Figure 6 illustrates these definitions by means of an example. Note that at this stage gaps are not modelled in our formulation. Hence, we have to extend our model to incorporate gap penalties in our model.

#### Gap edges

**Figure 7:**
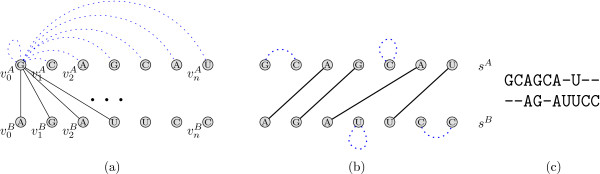
**Graph model augmented gap edges**. (a) Initial model additionally augmented with gap edges. The figure shows possible alignments edges and all gap edges starting from v0A
 MathType@MTEF@5@5@+=feaafiart1ev1aaatCvAUfKttLearuWrP9MDH5MBPbIqV92AaeXatLxBI9gBaebbnrfifHhDYfgasaacH8akY=wiFfYdH8Gipec8Eeeu0xXdbba9frFj0=OqFfea0dXdd9vqai=hGuQ8kuc9pgc9s8qqaq=dirpe0xb9q8qiLsFr0=vr0=vr0dc8meaabaqaciaacaGaaeqabaqabeGadaaakeaacqWG2bGDdaqhaaWcbaGaeGimaadabaGaemyqaeeaaaaa@3047@ (for sake of clarity, all other gap edges and interaction edges are not displayed). Note, however, that every node has outgoing gap edges to all other nodes in the sequence. The subset of lines and gap edges (b) corresponds to the alignment (c).

The initial model containing only lines (the set *L*) and interaction edges (the set *I*) is augmented by a set of *gap edges G*, which represents gaps in the alignment. For sake of compactness, we just describe the gap edges of sequence *A*, the gap edges of sequence *B *are defined analogously: We have an edge eklA
 MathType@MTEF@5@5@+=feaafiart1ev1aaatCvAUfKttLearuWrP9MDH5MBPbIqV92AaeXatLxBI9gBaebbnrfifHhDYfgasaacH8akY=wiFfYdH8Gipec8Eeeu0xXdbba9frFj0=OqFfea0dXdd9vqai=hGuQ8kuc9pgc9s8qqaq=dirpe0xb9q8qiLsFr0=vr0=vr0dc8meaabaqaciaacaGaaeqabaqabeGadaaakeaacqWGLbqzdaqhaaWcbaGaem4AaSMaemiBaWgabaGaemyqaeeaaaaa@31F7@ from vkA
 MathType@MTEF@5@5@+=feaafiart1ev1aaatCvAUfKttLearuWrP9MDH5MBPbIqV92AaeXatLxBI9gBaebbnrfifHhDYfgasaacH8akY=wiFfYdH8Gipec8Eeeu0xXdbba9frFj0=OqFfea0dXdd9vqai=hGuQ8kuc9pgc9s8qqaq=dirpe0xb9q8qiLsFr0=vr0=vr0dc8meaabaqaciaacaGaaeqabaqabeGadaaakeaacqWG2bGDdaqhaaWcbaGaem4AaSgabaGaemyqaeeaaaaa@30B8@ to vlA
 MathType@MTEF@5@5@+=feaafiart1ev1aaatCvAUfKttLearuWrP9MDH5MBPbIqV92AaeXatLxBI9gBaebbnrfifHhDYfgasaacH8akY=wiFfYdH8Gipec8Eeeu0xXdbba9frFj0=OqFfea0dXdd9vqai=hGuQ8kuc9pgc9s8qqaq=dirpe0xb9q8qiLsFr0=vr0=vr0dc8meaabaqaciaacaGaaeqabaqabeGadaaakeaacqWG2bGDdaqhaaWcbaGaemiBaWgabaGaemyqaeeaaaaa@30BA@ with *k*, *l *∈ 1, ..., |*s*^*A*^| representing the fact that no character of the substring skA...slA
 MathType@MTEF@5@5@+=feaafiart1ev1aaatCvAUfKttLearuWrP9MDH5MBPbIqV92AaeXatLxBI9gBaebbnrfifHhDYfgasaacH8akY=wiFfYdH8Gipec8Eeeu0xXdbba9frFj0=OqFfea0dXdd9vqai=hGuQ8kuc9pgc9s8qqaq=dirpe0xb9q8qiLsFr0=vr0=vr0dc8meaabaqaciaacaGaaeqabaqabeGadaaakeaacqWGZbWCdaqhaaWcbaGaem4AaSgabaGaemyqaeeaaOGaeiOla4IaeiOla4IaeiOla4Iaem4Cam3aa0baaSqaaiabdYgaSbqaaiabdgeabbaaaaa@3770@ is aligned to any character of the sequence *B*, whereas sk−1A
 MathType@MTEF@5@5@+=feaafiart1ev1aaatCvAUfKttLearuWrP9MDH5MBPbIqV92AaeXatLxBI9gBaebbnrfifHhDYfgasaacH8akY=wiFfYdH8Gipec8Eeeu0xXdbba9frFj0=OqFfea0dXdd9vqai=hGuQ8kuc9pgc9s8qqaq=dirpe0xb9q8qiLsFr0=vr0=vr0dc8meaabaqaciaacaGaaeqabaqabeGadaaakeaacqWGZbWCdaqhaaWcbaGaem4AaSMaeyOeI0IaeGymaedabaGaemyqaeeaaaaa@328F@ (if *k *- 1 > 0) and sl+1A
 MathType@MTEF@5@5@+=feaafiart1ev1aaatCvAUfKttLearuWrP9MDH5MBPbIqV92AaeXatLxBI9gBaebbnrfifHhDYfgasaacH8akY=wiFfYdH8Gipec8Eeeu0xXdbba9frFj0=OqFfea0dXdd9vqai=hGuQ8kuc9pgc9s8qqaq=dirpe0xb9q8qiLsFr0=vr0=vr0dc8meaabaqaciaacaGaaeqabaqabeGadaaakeaacqWGZbWCdaqhaaWcbaGaemiBaWMaey4kaSIaeGymaedabaGaemyqaeeaaaaa@3286@ (if *l *+ 1 ≤ |*s*^*B*^|) are aligned with some characters in sequence *B*. We say that vkA,...,vlA
 MathType@MTEF@5@5@+=feaafiart1ev1aaatCvAUfKttLearuWrP9MDH5MBPbIqV92AaeXatLxBI9gBaebbnrfifHhDYfgasaacH8akY=wiFfYdH8Gipec8Eeeu0xXdbba9frFj0=OqFfea0dXdd9vqai=hGuQ8kuc9pgc9s8qqaq=dirpe0xb9q8qiLsFr0=vr0=vr0dc8meaabaqaciaacaGaaeqabaqabeGadaaakeaacqWG2bGDdaqhaaWcbaGaem4AaSgabaGaemyqaeeaaOGaeiilaWIaeiOla4IaeiOla4IaeiOla4IaeiilaWIaemODay3aa0baaSqaaiabdYgaSbqaaiabdgeabbaaaaa@393C@ are *spanned *by the gap edge eklA
 MathType@MTEF@5@5@+=feaafiart1ev1aaatCvAUfKttLearuWrP9MDH5MBPbIqV92AaeXatLxBI9gBaebbnrfifHhDYfgasaacH8akY=wiFfYdH8Gipec8Eeeu0xXdbba9frFj0=OqFfea0dXdd9vqai=hGuQ8kuc9pgc9s8qqaq=dirpe0xb9q8qiLsFr0=vr0=vr0dc8meaabaqaciaacaGaaeqabaqabeGadaaakeaacqWGLbqzdaqhaaWcbaGaem4AaSMaemiBaWgabaGaemyqaeeaaaaa@31F7@. Figure 7 shows the graph extended by gap edges.

**Figure 8:**
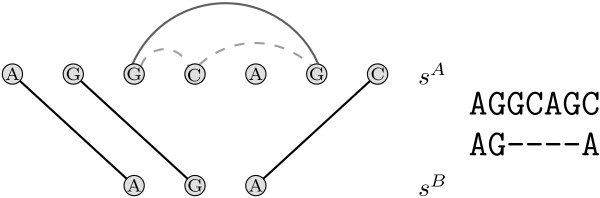
**Crossing gaps**. Gaps have to be realized by exactly one gap edge (in this example represented by the solid gray line), and cannot be split into two separate smaller gaps (the two dotted gap edges in this example).

Two gap edges eklA
 MathType@MTEF@5@5@+=feaafiart1ev1aaatCvAUfKttLearuWrP9MDH5MBPbIqV92AaeXatLxBI9gBaebbnrfifHhDYfgasaacH8akY=wiFfYdH8Gipec8Eeeu0xXdbba9frFj0=OqFfea0dXdd9vqai=hGuQ8kuc9pgc9s8qqaq=dirpe0xb9q8qiLsFr0=vr0=vr0dc8meaabaqaciaacaGaaeqabaqabeGadaaakeaacqWGLbqzdaqhaaWcbaGaem4AaSMaemiBaWgabaGaemyqaeeaaaaa@31F7@ and emnA
 MathType@MTEF@5@5@+=feaafiart1ev1aaatCvAUfKttLearuWrP9MDH5MBPbIqV92AaeXatLxBI9gBaebbnrfifHhDYfgasaacH8akY=wiFfYdH8Gipec8Eeeu0xXdbba9frFj0=OqFfea0dXdd9vqai=hGuQ8kuc9pgc9s8qqaq=dirpe0xb9q8qiLsFr0=vr0=vr0dc8meaabaqaciaacaGaaeqabaqabeGadaaakeaacqWGLbqzdaqhaaWcbaGaemyBa0MaemOBa4gabaGaemyqaeeaaaaa@31FF@ ∈ *G *are *in conflict *with each other if {*k*, ..., *l *+ 1} ∩ {*m*, ..., *n*} ≠ ∅, that is, if they overlap or touch. This is intuitively clear, because we do not want to split a longer gap into two separate gaps: Consequently, there has to be at least one aligned character between any two realized gap edges. See Fig. 8 for an example. We denote with the set CG
 MathType@MTEF@5@5@+=feaafiart1ev1aaatCvAUfKttLearuWrP9MDH5MBPbIqV92AaeXatLxBI9gBaebbnrfifHhDYfgasaacH8akY=wiFfYdH8Gipec8Eeeu0xXdbba9frFj0=OqFfea0dXdd9vqai=hGuQ8kuc9pgc9s8qqaq=dirpe0xb9q8qiLsFr0=vr0=vr0dc8meaabaqaciaacaGaaeqabaqabeGadaaakeaat0uy0HwzTfgDPnwy1egaryqtHrhAL1wy0L2yHvdaiqaacqWFce=qdaWgaaWcbaGaem4raCeabeaaaaa@3967@ the collection of all maximal sets of mutually conflicting gap edges. Finally, we define GvkA↔vlA
 MathType@MTEF@5@5@+=feaafiart1ev1aaatCvAUfKttLearuWrP9MDH5MBPbIqV92AaeXatLxBI9gBaebbnrfifHhDYfgasaacH8akY=wiFfYdH8Gipec8Eeeu0xXdbba9frFj0=OqFfea0dXdd9vqai=hGuQ8kuc9pgc9s8qqaq=dirpe0xb9q8qiLsFr0=vr0=vr0dc8meaabaqaciaacaGaaeqabaqabeGadaaakeaacqWGhbWrdaWgaaWcbaGaemODay3aa0baaWqaaiabdUgaRbqaaiabdgeabbaaliabgsziRkabdAha2naaDaaameaacqWGSbaBaeaacqWGbbqqaaaaleqaaaaa@380D@ as the set of gap edges that span the nodes vkA,...,vlA
 MathType@MTEF@5@5@+=feaafiart1ev1aaatCvAUfKttLearuWrP9MDH5MBPbIqV92AaeXatLxBI9gBaebbnrfifHhDYfgasaacH8akY=wiFfYdH8Gipec8Eeeu0xXdbba9frFj0=OqFfea0dXdd9vqai=hGuQ8kuc9pgc9s8qqaq=dirpe0xb9q8qiLsFr0=vr0=vr0dc8meaabaqaciaacaGaaeqabaqabeGadaaakeaacqWG2bGDdaqhaaWcbaGaem4AaSgabaGaemyqaeeaaOGaeiilaWIaeiOla4IaeiOla4IaeiOla4IaeiilaWIaemODay3aa0baaSqaaiabdYgaSbqaaiabdgeabbaaaaa@393C@.

#### Interaction match

**Figure 9:**
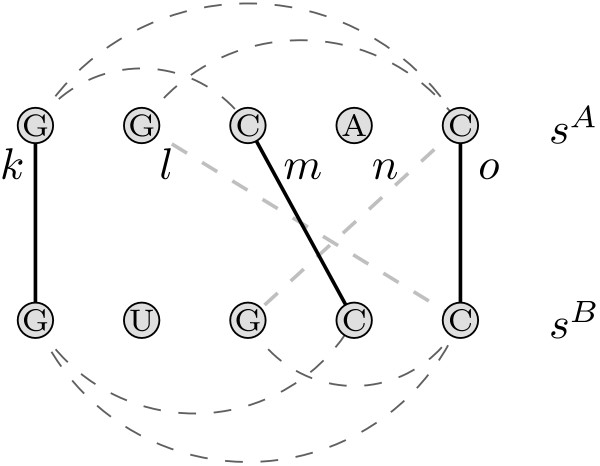
**Interaction match**. The pairs (*k*, *m*) and (*k*, *o*) are valid interaction matches. The pair (*l*, *n*), however, is not a valid interaction match since *l *and *n *cross each other.

We call two interactions (skA,slA)∈pA
 MathType@MTEF@5@5@+=feaafiart1ev1aaatCvAUfKttLearuWrP9MDH5MBPbIqV92AaeXatLxBI9gBaebbnrfifHhDYfgasaacH8akY=wiFfYdH8Gipec8Eeeu0xXdbba9frFj0=OqFfea0dXdd9vqai=hGuQ8kuc9pgc9s8qqaq=dirpe0xb9q8qiLsFr0=vr0=vr0dc8meaabaqaciaacaGaaeqabaqabeGadaaakeaacqGGOaakcqWGZbWCdaqhaaWcbaGaem4AaSgabaGaemyqaeeaaOGaeiilaWIaem4Cam3aa0baaSqaaiabdYgaSbqaaiabdgeabbaakiabcMcaPiabgIGiolabdchaWnaaCaaaleqabaGaemyqaeeaaaaa@3B85@ and (smB,snB)∈pB
 MathType@MTEF@5@5@+=feaafiart1ev1aaatCvAUfKttLearuWrP9MDH5MBPbIqV92AaeXatLxBI9gBaebbnrfifHhDYfgasaacH8akY=wiFfYdH8Gipec8Eeeu0xXdbba9frFj0=OqFfea0dXdd9vqai=hGuQ8kuc9pgc9s8qqaq=dirpe0xb9q8qiLsFr0=vr0=vr0dc8meaabaqaciaacaGaaeqabaqabeGadaaakeaacqGGOaakcqWGZbWCdaqhaaWcbaGaemyBa0gabaGaemOqaieaaOGaeiilaWIaem4Cam3aa0baaSqaaiabd6gaUbqaaiabdkeacbaakiabcMcaPiabgIGiolabdchaWnaaCaaaleqabaGaemOqaieaaaaa@3B93@ an *interaction match *if there exist two alignment edges a=(vkA,vmB)
 MathType@MTEF@5@5@+=feaafiart1ev1aaatCvAUfKttLearuWrP9MDH5MBPbIqV92AaeXatLxBI9gBaebbnrfifHhDYfgasaacH8akY=wiFfYdH8Gipec8Eeeu0xXdbba9frFj0=OqFfea0dXdd9vqai=hGuQ8kuc9pgc9s8qqaq=dirpe0xb9q8qiLsFr0=vr0=vr0dc8meaabaqaciaacaGaaeqabaqabeGadaaakeaacqWGHbqycqGH9aqpcqGGOaakcqWG2bGDdaqhaaWcbaGaem4AaSgabaGaemyqaeeaaOGaeiilaWIaemODay3aa0baaSqaaiabd2gaTbqaaiabdkeacbaakiabcMcaPaaa@39C1@ and b=(vlA,vnB)
 MathType@MTEF@5@5@+=feaafiart1ev1aaatCvAUfKttLearuWrP9MDH5MBPbIqV92AaeXatLxBI9gBaebbnrfifHhDYfgasaacH8akY=wiFfYdH8Gipec8Eeeu0xXdbba9frFj0=OqFfea0dXdd9vqai=hGuQ8kuc9pgc9s8qqaq=dirpe0xb9q8qiLsFr0=vr0=vr0dc8meaabaqaciaacaGaaeqabaqabeGadaaakeaacqWGIbGycqGH9aqpcqGGOaakcqWG2bGDdaqhaaWcbaGaemiBaWgabaGaemyqaeeaaOGaeiilaWIaemODay3aa0baaSqaaiabd6gaUbqaaiabdkeacbaakiabcMcaPaaa@39C7@ that do not cross each other. We say that a subset S
 MathType@MTEF@5@5@+=feaafiart1ev1aaatCvAUfKttLearuWrP9MDH5MBPbIqV92AaeXatLxBI9gBaebbnrfifHhDYfgasaacH8akY=wiFfYdH8Gipec8Eeeu0xXdbba9frFj0=OqFfea0dXdd9vqai=hGuQ8kuc9pgc9s8qqaq=dirpe0xb9q8qiLsFr0=vr0=vr0dc8meaabaqaciaacaGaaeqabaqabeGadaaakeaat0uy0HwzTfgDPnwy1egaryqtHrhAL1wy0L2yHvdaiqaacqWFse=uaaa@3844@ ⊆ *L realizes *the interaction match if {*a*, *b*} ⊆ S
 MathType@MTEF@5@5@+=feaafiart1ev1aaatCvAUfKttLearuWrP9MDH5MBPbIqV92AaeXatLxBI9gBaebbnrfifHhDYfgasaacH8akY=wiFfYdH8Gipec8Eeeu0xXdbba9frFj0=OqFfea0dXdd9vqai=hGuQ8kuc9pgc9s8qqaq=dirpe0xb9q8qiLsFr0=vr0=vr0dc8meaabaqaciaacaGaaeqabaqabeGadaaakeaat0uy0HwzTfgDPnwy1egaryqtHrhAL1wy0L2yHvdaiqaacqWFse=uaaa@3844@. Interaction matches realized by a set S
 MathType@MTEF@5@5@+=feaafiart1ev1aaatCvAUfKttLearuWrP9MDH5MBPbIqV92AaeXatLxBI9gBaebbnrfifHhDYfgasaacH8akY=wiFfYdH8Gipec8Eeeu0xXdbba9frFj0=OqFfea0dXdd9vqai=hGuQ8kuc9pgc9s8qqaq=dirpe0xb9q8qiLsFr0=vr0=vr0dc8meaabaqaciaacaGaaeqabaqabeGadaaakeaat0uy0HwzTfgDPnwy1egaryqtHrhAL1wy0L2yHvdaiqaacqWFse=uaaa@3844@ represent common interactions that are preserved by aligning the begin and end nucleotides of the interaction. Figure 9 illustrates the definitions.

#### Gapped structural trace

A triple (ℒ
 MathType@MTEF@5@5@+=feaafiart1ev1aaatCvAUfKttLearuWrP9MDH5MBPbIqV92AaeXatLxBI9gBaebbnrfifHhDYfgasaacH8akY=wiFfYdH8Gipec8Eeeu0xXdbba9frFj0=OqFfea0dXdd9vqai=hGuQ8kuc9pgc9s8qqaq=dirpe0xb9q8qiLsFr0=vr0=vr0dc8meaabaqaciaacaGaaeqabaqabeGadaaakeaat0uy0HwzTfgDPnwy1egaryqtHrhAL1wy0L2yHvdaiqaacqWFsectaaa@376D@, ℐ
 MathType@MTEF@5@5@+=feaafiart1ev1aaatCvAUfKttLearuWrP9MDH5MBPbIqV92AaeXatLxBI9gBaebbnrfifHhDYfgasaacH8akY=wiFfYdH8Gipec8Eeeu0xXdbba9frFj0=OqFfea0dXdd9vqai=hGuQ8kuc9pgc9s8qqaq=dirpe0xb9q8qiLsFr0=vr0=vr0dc8meaabaqaciaacaGaaeqabaqabeGadaaakeaat0uy0HwzTfgDPnwy1egaryqtHrhAL1wy0L2yHvdaiqaacqWFqessaaa@3768@, G
 MathType@MTEF@5@5@+=feaafiart1ev1aaatCvAUfKttLearuWrP9MDH5MBPbIqV92AaeXatLxBI9gBaebbnrfifHhDYfgasaacH8akY=wiFfYdH8Gipec8Eeeu0xXdbba9frFj0=OqFfea0dXdd9vqai=hGuQ8kuc9pgc9s8qqaq=dirpe0xb9q8qiLsFr0=vr0=vr0dc8meaabaqaciaacaGaaeqabaqabeGadaaakeaat0uy0HwzTfgDPnwy1egaryqtHrhAL1wy0L2yHvdaiqaacqWFge=raaa@382C@) with ℒ
 MathType@MTEF@5@5@+=feaafiart1ev1aaatCvAUfKttLearuWrP9MDH5MBPbIqV92AaeXatLxBI9gBaebbnrfifHhDYfgasaacH8akY=wiFfYdH8Gipec8Eeeu0xXdbba9frFj0=OqFfea0dXdd9vqai=hGuQ8kuc9pgc9s8qqaq=dirpe0xb9q8qiLsFr0=vr0=vr0dc8meaabaqaciaacaGaaeqabaqabeGadaaakeaat0uy0HwzTfgDPnwy1egaryqtHrhAL1wy0L2yHvdaiqaacqWFsectaaa@376D@ ⊆ *L*, ℐ
 MathType@MTEF@5@5@+=feaafiart1ev1aaatCvAUfKttLearuWrP9MDH5MBPbIqV92AaeXatLxBI9gBaebbnrfifHhDYfgasaacH8akY=wiFfYdH8Gipec8Eeeu0xXdbba9frFj0=OqFfea0dXdd9vqai=hGuQ8kuc9pgc9s8qqaq=dirpe0xb9q8qiLsFr0=vr0=vr0dc8meaabaqaciaacaGaaeqabaqabeGadaaakeaat0uy0HwzTfgDPnwy1egaryqtHrhAL1wy0L2yHvdaiqaacqWFqessaaa@3768@ ⊆ *I*, and G
 MathType@MTEF@5@5@+=feaafiart1ev1aaatCvAUfKttLearuWrP9MDH5MBPbIqV92AaeXatLxBI9gBaebbnrfifHhDYfgasaacH8akY=wiFfYdH8Gipec8Eeeu0xXdbba9frFj0=OqFfea0dXdd9vqai=hGuQ8kuc9pgc9s8qqaq=dirpe0xb9q8qiLsFr0=vr0=vr0dc8meaabaqaciaacaGaaeqabaqabeGadaaakeaat0uy0HwzTfgDPnwy1egaryqtHrhAL1wy0L2yHvdaiqaacqWFge=raaa@382C@ ⊆ *G *is called a valid *gapped structural trace *if and only if the following constraints are satisfied:

1. The vertices vlA
 MathType@MTEF@5@5@+=feaafiart1ev1aaatCvAUfKttLearuWrP9MDH5MBPbIqV92AaeXatLxBI9gBaebbnrfifHhDYfgasaacH8akY=wiFfYdH8Gipec8Eeeu0xXdbba9frFj0=OqFfea0dXdd9vqai=hGuQ8kuc9pgc9s8qqaq=dirpe0xb9q8qiLsFr0=vr0=vr0dc8meaabaqaciaacaGaaeqabaqabeGadaaakeaacqWG2bGDdaqhaaWcbaGaemiBaWgabaGaemyqaeeaaaaa@30BA@ and vkB
 MathType@MTEF@5@5@+=feaafiart1ev1aaatCvAUfKttLearuWrP9MDH5MBPbIqV92AaeXatLxBI9gBaebbnrfifHhDYfgasaacH8akY=wiFfYdH8Gipec8Eeeu0xXdbba9frFj0=OqFfea0dXdd9vqai=hGuQ8kuc9pgc9s8qqaq=dirpe0xb9q8qiLsFr0=vr0=vr0dc8meaabaqaciaacaGaaeqabaqabeGadaaakeaacqWG2bGDdaqhaaWcbaGaem4AaSgabaGaemOqaieaaaaa@30BA@ of sequences *A *and *B *are either incident to exactly one alignment edge *e *∈ ℒ
 MathType@MTEF@5@5@+=feaafiart1ev1aaatCvAUfKttLearuWrP9MDH5MBPbIqV92AaeXatLxBI9gBaebbnrfifHhDYfgasaacH8akY=wiFfYdH8Gipec8Eeeu0xXdbba9frFj0=OqFfea0dXdd9vqai=hGuQ8kuc9pgc9s8qqaq=dirpe0xb9q8qiLsFr0=vr0=vr0dc8meaabaqaciaacaGaaeqabaqabeGadaaakeaat0uy0HwzTfgDPnwy1egaryqtHrhAL1wy0L2yHvdaiqaacqWFsectaaa@376D@ or spanned by a gap edge *g *∈ G
 MathType@MTEF@5@5@+=feaafiart1ev1aaatCvAUfKttLearuWrP9MDH5MBPbIqV92AaeXatLxBI9gBaebbnrfifHhDYfgasaacH8akY=wiFfYdH8Gipec8Eeeu0xXdbba9frFj0=OqFfea0dXdd9vqai=hGuQ8kuc9pgc9s8qqaq=dirpe0xb9q8qiLsFr0=vr0=vr0dc8meaabaqaciaacaGaaeqabaqabeGadaaakeaat0uy0HwzTfgDPnwy1egaryqtHrhAL1wy0L2yHvdaiqaacqWFge=raaa@382C@. In other words, a nucleotide is either aligned or "aligned" to a gap.

2. A line *l *can realize at most one interaction match (*l*, *m*), because a nucleotide can pair with at most one other nucleotide in a valid RNA secondary structure.

3. There are no two lines *k*, *l *∈ *L *that cross or touch each other: Crossing lines induce ordering conflicts in the alignment, whereas touching lines imply that two different nucleotides are mapped to the same nucleotide in the other sequence.

4. There are no two gaps edges eklA,emnA∈G
 MathType@MTEF@5@5@+=feaafiart1ev1aaatCvAUfKttLearuWrP9MDH5MBPbIqV92AaeXatLxBI9gBaebbnrfifHhDYfgasaacH8akY=wiFfYdH8Gipec8Eeeu0xXdbba9frFj0=OqFfea0dXdd9vqai=hGuQ8kuc9pgc9s8qqaq=dirpe0xb9q8qiLsFr0=vr0=vr0dc8meaabaqaciaacaGaaeqabaqabeGadaaakeaacqWGLbqzdaqhaaWcbaGaem4AaSMaemiBaWgabaGaemyqaeeaaOGaeiilaWIaemyzau2aa0baaSqaaiabd2gaTjabd6gaUbqaaiabdgeabbaakiabgIGioprtHrhAL1wy0L2yHvtyaeHbnfgDOvwBHrxAJfwnaGabaiab=zq8hbaa@4542@ such that eklA
 MathType@MTEF@5@5@+=feaafiart1ev1aaatCvAUfKttLearuWrP9MDH5MBPbIqV92AaeXatLxBI9gBaebbnrfifHhDYfgasaacH8akY=wiFfYdH8Gipec8Eeeu0xXdbba9frFj0=OqFfea0dXdd9vqai=hGuQ8kuc9pgc9s8qqaq=dirpe0xb9q8qiLsFr0=vr0=vr0dc8meaabaqaciaacaGaaeqabaqabeGadaaakeaacqWGLbqzdaqhaaWcbaGaem4AaSMaemiBaWgabaGaemyqaeeaaaaa@31F7@ is in conflict with emnA
 MathType@MTEF@5@5@+=feaafiart1ev1aaatCvAUfKttLearuWrP9MDH5MBPbIqV92AaeXatLxBI9gBaebbnrfifHhDYfgasaacH8akY=wiFfYdH8Gipec8Eeeu0xXdbba9frFj0=OqFfea0dXdd9vqai=hGuQ8kuc9pgc9s8qqaq=dirpe0xb9q8qiLsFr0=vr0=vr0dc8meaabaqaciaacaGaaeqabaqabeGadaaakeaacqWGLbqzdaqhaaWcbaGaemyBa0MaemOBa4gabaGaemyqaeeaaaaa@31FF@, and there are no two gaps edges eklB,emnB∈G
 MathType@MTEF@5@5@+=feaafiart1ev1aaatCvAUfKttLearuWrP9MDH5MBPbIqV92AaeXatLxBI9gBaebbnrfifHhDYfgasaacH8akY=wiFfYdH8Gipec8Eeeu0xXdbba9frFj0=OqFfea0dXdd9vqai=hGuQ8kuc9pgc9s8qqaq=dirpe0xb9q8qiLsFr0=vr0=vr0dc8meaabaqaciaacaGaaeqabaqabeGadaaakeaacqWGLbqzdaqhaaWcbaGaem4AaSMaemiBaWgabaGaemOqaieaaOGaeiilaWIaemyzau2aa0baaSqaaiabd2gaTjabd6gaUbqaaiabdkeacbaakiabgIGioprtHrhAL1wy0L2yHvtyaeHbnfgDOvwBHrxAJfwnaGabaiab=zq8hbaa@4546@ such that eklB
 MathType@MTEF@5@5@+=feaafiart1ev1aaatCvAUfKttLearuWrP9MDH5MBPbIqV92AaeXatLxBI9gBaebbnrfifHhDYfgasaacH8akY=wiFfYdH8Gipec8Eeeu0xXdbba9frFj0=OqFfea0dXdd9vqai=hGuQ8kuc9pgc9s8qqaq=dirpe0xb9q8qiLsFr0=vr0=vr0dc8meaabaqaciaacaGaaeqabaqabeGadaaakeaacqWGLbqzdaqhaaWcbaGaem4AaSMaemiBaWgabaGaemOqaieaaaaa@31F9@ is in conflict with emnB
 MathType@MTEF@5@5@+=feaafiart1ev1aaatCvAUfKttLearuWrP9MDH5MBPbIqV92AaeXatLxBI9gBaebbnrfifHhDYfgasaacH8akY=wiFfYdH8Gipec8Eeeu0xXdbba9frFj0=OqFfea0dXdd9vqai=hGuQ8kuc9pgc9s8qqaq=dirpe0xb9q8qiLsFr0=vr0=vr0dc8meaabaqaciaacaGaaeqabaqabeGadaaakeaacqWGLbqzdaqhaaWcbaGaemyBa0MaemOBa4gabaGaemOqaieaaaaa@3201@.

**Figure 10:**
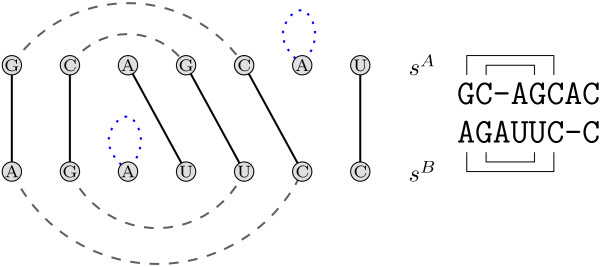
**Valid gapped structural trace**. Valid gapped structural trace: every vertex is incident to exactly one line or is spanned by a gap edge. There are no crossing lines, and every line is incident to at most one interaction match.

Figure 10 visualizes these properties by showing a toy example for a gapped structural trace.

We assign weights *w*_*l *_and *w*_*kl *_for each line *l *and interaction match (*k*, *l*) that represents the benefit of realizing *l *or (*k*, *l*). By default, we set these scores along the lines of standard scoring methods, e.g., BLOSUM matrices for the weight of the lines, *base pair probabilities *[18] for the interaction match scores, or by using the RIBOSUM scoring matrices derived from alignments of ribosomal RNAs [47]. Our model, however, is not limited to standard scoring schemes. Since we can set each (sequence or structure) weight separately, the user can assign completely arbitrary scores to each line or interaction match which makes the incorporation of expert knowledge into the computation of structural alignments easy. Furthermore, we assign negative weights to gap edges aklA
 MathType@MTEF@5@5@+=feaafiart1ev1aaatCvAUfKttLearuWrP9MDH5MBPbIqV92AaeXatLxBI9gBaebbnrfifHhDYfgasaacH8akY=wiFfYdH8Gipec8Eeeu0xXdbba9frFj0=OqFfea0dXdd9vqai=hGuQ8kuc9pgc9s8qqaq=dirpe0xb9q8qiLsFr0=vr0=vr0dc8meaabaqaciaacaGaaeqabaqabeGadaaakeaacqWGHbqydaqhaaWcbaGaem4AaSMaemiBaWgabaGaemyqaeeaaaaa@31EF@ with representing the gap penalty for aligning substring skA,...,slA
 MathType@MTEF@5@5@+=feaafiart1ev1aaatCvAUfKttLearuWrP9MDH5MBPbIqV92AaeXatLxBI9gBaebbnrfifHhDYfgasaacH8akY=wiFfYdH8Gipec8Eeeu0xXdbba9frFj0=OqFfea0dXdd9vqai=hGuQ8kuc9pgc9s8qqaq=dirpe0xb9q8qiLsFr0=vr0=vr0dc8meaabaqaciaacaGaaeqabaqabeGadaaakeaacqWGZbWCdaqhaaWcbaGaem4AaSgabaGaemyqaeeaaOGaeiilaWIaeiOla4IaeiOla4IaeiOla4IaeiilaWIaem4Cam3aa0baaSqaaiabdYgaSbqaaiabdgeabbaaaaa@3930@ with gap characters. Note that the model allows for arbitrary, position-dependent gap scoring.

Approaches for traditional sequence alignment aim at maximizing the score of edges in an alignment ℒ
 MathType@MTEF@5@5@+=feaafiart1ev1aaatCvAUfKttLearuWrP9MDH5MBPbIqV92AaeXatLxBI9gBaebbnrfifHhDYfgasaacH8akY=wiFfYdH8Gipec8Eeeu0xXdbba9frFj0=OqFfea0dXdd9vqai=hGuQ8kuc9pgc9s8qqaq=dirpe0xb9q8qiLsFr0=vr0=vr0dc8meaabaqaciaacaGaaeqabaqabeGadaaakeaat0uy0HwzTfgDPnwy1egaryqtHrhAL1wy0L2yHvdaiqaacqWFsectaaa@376D@. Structural alignments, however, must also take the structural information encoded in the interaction edges into account. The problem of structurally aligning two annotated sequences (*s*^*A*^, *p*^*A*^) and (*s*^*B*^, *p*^*B*^) corresponds to finding an alignment such that the weight of the sequence part (i.e., the weight of selected lines plus gap penalties) plus the weight of the realized interaction matches is maximal. More formally, we seek to maximize ∑l∈ℒwl+∑g∈Gwg+∑(i,j)∈ℐwij
 MathType@MTEF@5@5@+=feaafiart1ev1aaatCvAUfKttLearuWrP9MDH5MBPbIqV92AaeXatLxBI9gBaebbnrfifHhDYfgasaacH8akY=wiFfYdH8Gipec8Eeeu0xXdbba9frFj0=OqFfea0dXdd9vqai=hGuQ8kuc9pgc9s8qqaq=dirpe0xb9q8qiLsFr0=vr0=vr0dc8meaabaqaciaacaGaaeqabaqabeGadaaakeaadaaeqaqaaiabdEha3naaBaaaleaacqWGSbaBaeqaaaqaaiabdYgaSjabgIGioprtHrhAL1wy0L2yHvtyaeHbnfgDOvwBHrxAJfwnaGabaiab=jrimbqab0GaeyyeIuoakiabgUcaRmaaqababaGaem4DaC3aaSbaaSqaaiabdEgaNbqabaaabaGaem4zaCMaeyicI4Sae8NbXFeabeqdcqGHris5aOGaey4kaSYaaabeaeaacqWG3bWDdaWgaaWcbaGaemyAaKMaemOAaOgabeaaaeaacqGGOaakcqWGPbqAcqGGSaalcqWGQbGAcqGGPaqkcqGHiiIZcqWFqessaeqaniabggHiLdaaaa@5876@, where (ℒ
 MathType@MTEF@5@5@+=feaafiart1ev1aaatCvAUfKttLearuWrP9MDH5MBPbIqV92AaeXatLxBI9gBaebbnrfifHhDYfgasaacH8akY=wiFfYdH8Gipec8Eeeu0xXdbba9frFj0=OqFfea0dXdd9vqai=hGuQ8kuc9pgc9s8qqaq=dirpe0xb9q8qiLsFr0=vr0=vr0dc8meaabaqaciaacaGaaeqabaqabeGadaaakeaat0uy0HwzTfgDPnwy1egaryqtHrhAL1wy0L2yHvdaiqaacqWFsectaaa@376D@, G
 MathType@MTEF@5@5@+=feaafiart1ev1aaatCvAUfKttLearuWrP9MDH5MBPbIqV92AaeXatLxBI9gBaebbnrfifHhDYfgasaacH8akY=wiFfYdH8Gipec8Eeeu0xXdbba9frFj0=OqFfea0dXdd9vqai=hGuQ8kuc9pgc9s8qqaq=dirpe0xb9q8qiLsFr0=vr0=vr0dc8meaabaqaciaacaGaaeqabaqabeGadaaakeaat0uy0HwzTfgDPnwy1egaryqtHrhAL1wy0L2yHvdaiqaacqWFge=raaa@382C@) represents an alignment with arbitrary gap costs, and ℐ
 MathType@MTEF@5@5@+=feaafiart1ev1aaatCvAUfKttLearuWrP9MDH5MBPbIqV92AaeXatLxBI9gBaebbnrfifHhDYfgasaacH8akY=wiFfYdH8Gipec8Eeeu0xXdbba9frFj0=OqFfea0dXdd9vqai=hGuQ8kuc9pgc9s8qqaq=dirpe0xb9q8qiLsFr0=vr0=vr0dc8meaabaqaciaacaGaaeqabaqabeGadaaakeaat0uy0HwzTfgDPnwy1egaryqtHrhAL1wy0L2yHvdaiqaacqWFqessaaa@3768@ contains the interaction matches realized by ℒ
 MathType@MTEF@5@5@+=feaafiart1ev1aaatCvAUfKttLearuWrP9MDH5MBPbIqV92AaeXatLxBI9gBaebbnrfifHhDYfgasaacH8akY=wiFfYdH8Gipec8Eeeu0xXdbba9frFj0=OqFfea0dXdd9vqai=hGuQ8kuc9pgc9s8qqaq=dirpe0xb9q8qiLsFr0=vr0=vr0dc8meaabaqaciaacaGaaeqabaqabeGadaaakeaat0uy0HwzTfgDPnwy1egaryqtHrhAL1wy0L2yHvdaiqaacqWFsectaaa@376D@. Observe that this graph-theoretical reformulation matches the problem statement given at the beginning of this section.

#### Biological aspects

The basic entities of our model are the alignment, interaction, and gap edges in the structural graph, which contribute to the objective function rather independently. Hence, one could argue that the model does not capture important features of RNA structures, like the incorporation of stacking energies or loop scores that depend on the actual size of the loop. We are aware of these limitations.

Nevertheless, the results of our computational experiments presented in Sect. 3 show that this approach yields high-quality structural alignments. In the pairwise case, our graph-based model is competitive with state-of-the-art approaches and develops its strength with an increasing number of sequences, outperforming all other programs that we tested (for details see Sect. 3). Additionally, the authors of [48] showed that models that do not capture stacking energies and loops are still competitive.

Beyond, our graph-based approach offers the possibility to change the model from nucleotides as the working entities to stems: Instead of taking single nucleotides as the vertices of the structural graph, we could search for *candidate stems *in the sequences and introduce a vertex for each half-stem. This would allow us to incorporate energy-based scoring into our model, which then, however, will have to be adapted to take into account overlapping stem candidates.

### 2.2 Integer linear program and Lagrangian relaxation

Given the graph-theoretical model it is straightforward to transform it to an *integer linear program *(ILP). We associate binary variables with each line, interaction match, and gap edge, and model the constraints of a valid gapped structural trace by adding inequalities to the linear program.

The handling of lines and gap edges is straightforward: We associate a *x *and *z *variable to each line and gap edge, respectively. We set *x*_*l *_= 1 if and only if line *l *∈ *L *is part of the alignment ℒ
 MathType@MTEF@5@5@+=feaafiart1ev1aaatCvAUfKttLearuWrP9MDH5MBPbIqV92AaeXatLxBI9gBaebbnrfifHhDYfgasaacH8akY=wiFfYdH8Gipec8Eeeu0xXdbba9frFj0=OqFfea0dXdd9vqai=hGuQ8kuc9pgc9s8qqaq=dirpe0xb9q8qiLsFr0=vr0=vr0dc8meaabaqaciaacaGaaeqabaqabeGadaaakeaat0uy0HwzTfgDPnwy1egaryqtHrhAL1wy0L2yHvdaiqaacqWFsectaaa@376D@, and *z*_*a *_= 1 if and only if gap edge *a *∈ *G *is part of the alignment.

**Figure 11:**
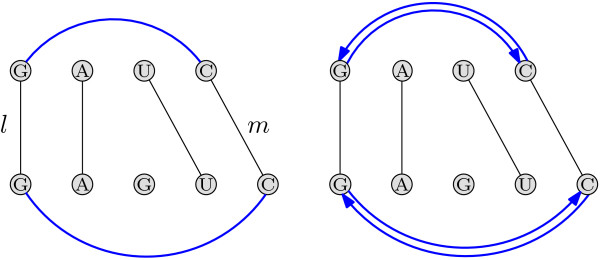
**Splitting of interaction matches**. One interaction match is split into two *directed *interaction matches.

Interaction matches, however, are treated slightly differently: Instead of assigning an ILP variable to each interaction edge, we split an interaction match (*l*, *m*) into two separate *directed interaction matches *(*l*, *m*) and (*m, l*) that are detached from each other. A directed interaction match (*l*, *m*) is *realized *by the line set ℒ
 MathType@MTEF@5@5@+=feaafiart1ev1aaatCvAUfKttLearuWrP9MDH5MBPbIqV92AaeXatLxBI9gBaebbnrfifHhDYfgasaacH8akY=wiFfYdH8Gipec8Eeeu0xXdbba9frFj0=OqFfea0dXdd9vqai=hGuQ8kuc9pgc9s8qqaq=dirpe0xb9q8qiLsFr0=vr0=vr0dc8meaabaqaciaacaGaaeqabaqabeGadaaakeaat0uy0HwzTfgDPnwy1egaryqtHrhAL1wy0L2yHvdaiqaacqWFsectaaa@376D@ if *l *∈ ℒ
 MathType@MTEF@5@5@+=feaafiart1ev1aaatCvAUfKttLearuWrP9MDH5MBPbIqV92AaeXatLxBI9gBaebbnrfifHhDYfgasaacH8akY=wiFfYdH8Gipec8Eeeu0xXdbba9frFj0=OqFfea0dXdd9vqai=hGuQ8kuc9pgc9s8qqaq=dirpe0xb9q8qiLsFr0=vr0=vr0dc8meaabaqaciaacaGaaeqabaqabeGadaaakeaat0uy0HwzTfgDPnwy1egaryqtHrhAL1wy0L2yHvdaiqaacqWFsectaaa@376D@. We then have *y*_*lm *_= 1 if and only if the directed interaction match (*l*, *m*) is realized (note again that *y*_*lm *_and *y*_*ml *_are distinct variables). Figure 11 gives an illustration of the variable splitting. Note that this does not change the underlying model, it just makes the ILP formulation more convenient for further processing. Splitting interaction matches has first been proposed by Caprara and Lancia in the context of contact map overlap [42].

As described in Sect. 2.1, the sets *L*, *I*, and *G *refer to lines, interaction edges, and gap edges, and the sets CL
 MathType@MTEF@5@5@+=feaafiart1ev1aaatCvAUfKttLearuWrP9MDH5MBPbIqV92AaeXatLxBI9gBaebbnrfifHhDYfgasaacH8akY=wiFfYdH8Gipec8Eeeu0xXdbba9frFj0=OqFfea0dXdd9vqai=hGuQ8kuc9pgc9s8qqaq=dirpe0xb9q8qiLsFr0=vr0=vr0dc8meaabaqaciaacaGaaeqabaqabeGadaaakeaat0uy0HwzTfgDPnwy1egaryqtHrhAL1wy0L2yHvdaiqaacqWFce=qdaWgaaWcbaGaemitaWeabeaaaaa@3971@ and CG
 MathType@MTEF@5@5@+=feaafiart1ev1aaatCvAUfKttLearuWrP9MDH5MBPbIqV92AaeXatLxBI9gBaebbnrfifHhDYfgasaacH8akY=wiFfYdH8Gipec8Eeeu0xXdbba9frFj0=OqFfea0dXdd9vqai=hGuQ8kuc9pgc9s8qqaq=dirpe0xb9q8qiLsFr0=vr0=vr0dc8meaabaqaciaacaGaaeqabaqabeGadaaakeaat0uy0HwzTfgDPnwy1egaryqtHrhAL1wy0L2yHvdaiqaacqWFce=qdaWgaaWcbaGaem4raCeabeaaaaa@3967@ contain subsets of mutually conflicting lines or gap edges.

We then give the following ILP formulation for the gapped structural trace problem:

max⁡∑l∈Lwlxl+∑l∈L∑m∈Lwlmylm+∑g∈Gwgzg
 MathType@MTEF@5@5@+=feaafiart1ev1aaatCvAUfKttLearuWrP9MDH5MBPbIqV92AaeXatLxBI9gBaebbnrfifHhDYfgasaacH8akY=wiFfYdH8Gipec8Eeeu0xXdbba9frFj0=OqFfea0dXdd9vqai=hGuQ8kuc9pgc9s8qqaq=dirpe0xb9q8qiLsFr0=vr0=vr0dc8meaabaqaciaacaGaaeqabaqabeGadaaakeaacyGGTbqBcqGGHbqycqGG4baEdaaeqbqaaiabdEha3naaBaaaleaacqWGSbaBaeqaaOGaemiEaG3aaSbaaSqaaiabdYgaSbqabaaabaGaemiBaWMaeyicI4SaemitaWeabeqdcqGHris5aOGaey4kaSYaaabuaeaadaaeqbqaaiabdEha3naaBaaaleaacqWGSbaBcqWGTbqBaeqaaOGaemyEaK3aaSbaaSqaaiabdYgaSjabd2gaTbqabaGccqGHRaWkdaaeqbqaaiabdEha3naaBaaaleaacqWGNbWzaeqaaaqaaiabdEgaNjabgIGiolabdEeahbqab0GaeyyeIuoakiabdQha6naaBaaaleaacqWGNbWzaeqaaaqaaiabd2gaTjabgIGiolabdYeambqab0GaeyyeIuoaaSqaaiabdYgaSjabgIGiolabdYeambqab0GaeyyeIuoaaaa@600F@

s.t.∑l∈CLxl≤1∀CL∈CL
 MathType@MTEF@5@5@+=feaafiart1ev1aaatCvAUfKttLearuWrP9MDH5MBPbIqV92AaeXatLxBI9gBaebbnrfifHhDYfgasaacH8akY=wiFfYdH8Gipec8Eeeu0xXdbba9frFj0=OqFfea0dXdd9vqai=hGuQ8kuc9pgc9s8qqaq=dirpe0xb9q8qiLsFr0=vr0=vr0dc8meaabaqaciaacaGaaeqabaqabeGadaaakeaafaqaaeqacaaabaacbaGae83Cam3exLMBbXgBcf2CPn2qVrwzqf2zLnharyavP1wzZbItLDhis9wBH5gaiqaacaGFUaGae8hDaqNaa4NlamaaqafabaGaemiEaG3aaSbaaSqaaiabdYgaSbqabaGccqGHKjYOcqaIXaqmaSqaaiabdYgaSjabgIGiolabdoeadnaaBaaameaacqWGmbataeqaaaWcbeqdcqGHris5aaGcbaGaeyiaIiIaem4qam0aaSbaaSqaaiabdYeambqabaGccqGHiiIZt0uy0HwzTfgDPnwy1egarCqtHrhAL1wy0L2yHvdaiuaacqqFce=qdaWgaaWcbaGaemitaWeabeaaaaaaaa@5CD6@

∑a∈CGza≤1∀CG∈CG
 MathType@MTEF@5@5@+=feaafiart1ev1aaatCvAUfKttLearuWrP9MDH5MBPbIqV92AaeXatLxBI9gBaebbnrfifHhDYfgasaacH8akY=wiFfYdH8Gipec8Eeeu0xXdbba9frFj0=OqFfea0dXdd9vqai=hGuQ8kuc9pgc9s8qqaq=dirpe0xb9q8qiLsFr0=vr0=vr0dc8meaabaqaciaacaGaaeqabaqabeGadaaakeaafaqaaeqacaaabaWaaabuaeaacqWG6bGEdaWgaaWcbaGaemyyaegabeaakiabgsMiJkabigdaXaWcbaGaemyyaeMaeyicI4Saem4qam0aaSbaaWqaaiabdEeahbqabaaaleqaniabggHiLdaakeaacqGHaiIicqWGdbWqdaWgaaWcbaGaem4raCeabeaakiabgIGioprtHrhAL1wy0L2yHvtyaeHbnfgDOvwBHrxAJfwnaGabaiab=jq8dnaaBaaaleaacqWGhbWraeqaaaaaaaa@4B20@

xl+∑a∈Gs(l)↔s(l)za=1∀l∈L
 MathType@MTEF@5@5@+=feaafiart1ev1aaatCvAUfKttLearuWrP9MDH5MBPbIqV92AaeXatLxBI9gBaebbnrfifHhDYfgasaacH8akY=wiFfYdH8Gipec8Eeeu0xXdbba9frFj0=OqFfea0dXdd9vqai=hGuQ8kuc9pgc9s8qqaq=dirpe0xb9q8qiLsFr0=vr0=vr0dc8meaabaqaciaacaGaaeqabaqabeGadaaakeaacqWG4baEdaWgaaWcbaGaemiBaWgabeaakiabgUcaRuaabaqabiaaaeaadaaeqbqaaiabdQha6naaBaaaleaacqWGHbqyaeqaaOGaeyypa0JaeGymaedaleaacqWGHbqycqGHiiIZcqWGhbWrdaWgaaadbaGaem4CamNaeiikaGIaemiBaWMaeiykaKIaeyiLHSQaem4CamNaeiikaGIaemiBaWMaeiykaKcabeaaaSqab0GaeyyeIuoaaOqaaiabgcGiIiabdYgaSjabgIGiolabdYeambaaaaa@4BAF@

xl+∑a∈Gt(l)↔t(l)za=1∀l∈L
 MathType@MTEF@5@5@+=feaafiart1ev1aaatCvAUfKttLearuWrP9MDH5MBPbIqV92AaeXatLxBI9gBaebbnrfifHhDYfgasaacH8akY=wiFfYdH8Gipec8Eeeu0xXdbba9frFj0=OqFfea0dXdd9vqai=hGuQ8kuc9pgc9s8qqaq=dirpe0xb9q8qiLsFr0=vr0=vr0dc8meaabaqaciaacaGaaeqabaqabeGadaaakeaacqWG4baEdaWgaaWcbaGaemiBaWgabeaakiabgUcaRuaabaqabiaaaeaadaaeqbqaaiabdQha6naaBaaaleaacqWGHbqyaeqaaOGaeyypa0JaeGymaedaleaacqWGHbqycqGHiiIZcqWGhbWrdaWgaaadbaGaemiDaqNaeiikaGIaemiBaWMaeiykaKIaeyiLHSQaemiDaqNaeiikaGIaemiBaWMaeiykaKcabeaaaSqab0GaeyyeIuoaaOqaaiabgcGiIiabdYgaSjabgIGiolabdYeambaaaaa@4BB3@

∑m∈Lylm≤xl∀l∈L
 MathType@MTEF@5@5@+=feaafiart1ev1aaatCvAUfKttLearuWrP9MDH5MBPbIqV92AaeXatLxBI9gBaebbnrfifHhDYfgasaacH8akY=wiFfYdH8Gipec8Eeeu0xXdbba9frFj0=OqFfea0dXdd9vqai=hGuQ8kuc9pgc9s8qqaq=dirpe0xb9q8qiLsFr0=vr0=vr0dc8meaabaqaciaacaGaaeqabaqabeGadaaakeaafaqaaeqacaaabaWaaabuaeaacqWG5bqEdaWgaaWcbaGaemiBaWMaemyBa0gabeaakiabgsMiJkabdIha4naaBaaaleaacqWGSbaBaeqaaaqaaiabd2gaTjabgIGiolabdYeambqab0GaeyyeIuoaaOqaaiabgcGiIiabdYgaSjabgIGiolabdYeambaaaaa@40E8@

*y*_*lm *_= *y*_*ml *_    ∀*l*, *m *∈ *L*

*x *∈ {0, 1}^*L *^*y *∈ {0, 1}^*L*×*L *^*z *∈ {0, 1}^*G*^

#### Lemma 2.1 (Proof in [16])

*A feasible solution to the ILP *(1)–(8) *corresponds to a valid gapped structural trace of weight equal to the objective function and vice versa*.

Observe that constraints (2)–(6) exactly correspond to the properties of a gapped structural trace as described in Sect. 2.1.

In [49] the authors show that the problem of computing an optimal gapped structural trace is already NP-hard, even without considering gap costs. Hence, we cannot hope to find an optimal solution to the problem in polynomial time.

Commonly used mathematical programming techniques for NP-hard problems therefore resort to various *relaxation techniques *that are the basis for further processing. A relaxation results from the removal of constraints from the original ILP formulation, and is often solvable in polynomial time. A popular relaxation is the so called *LP relaxation *where the integrality constraints on the variables are dropped, yielding a standard linear program, for which solutions can be found efficiently.

Another possible relaxation technique is *Lagrangian relaxation*: Instead of just dropping certain inequalities, we move them to the objective function, associated with a penalty term that becomes active if the dropped constraint is violated. By iteratively adapting those penalty terms using, for instance, *subgradient optimization*, we get better solutions with each iteration. A crucial parameter is therefore the number of iterations that we perform: the higher the number, the more likely it is to end up with an optimal or near-optimal solution.

Inspired by the successful approach of Lancia and Caprara for the contact map overlap problem, we consider the relaxation resulting from moving constraint (7) into the objective function.

#### Lemma 2.2 (Proof in [16])

*The relaxed problem is equivalent to the pairwise sequence alignment problem with arbitrary gap costs*.

### 2.3 Algorithms for the pairwise and multiple case

Our algorithm for the pairwise RNA structural alignment problem consists of iteratively solving the primary sequence alignment problem associated with the relaxation. The penalization of the relaxed inequality is reflected in an adapted scoring matrix for the primary alignment. Intuitively, these weights incorporate also the structural information. In each iteration we get a new lower bound for the problem by analyzing the primary sequence alignments and inferring the best *structural completion *of this alignment. In fact, this corresponds to solving a maximum weighted matching problem in a general graph. For details see [16]. In the course of the algorithm, these solutions get better and better. Furthermore, the value of the relaxation itself constitutes an upper bound on the problem, which decreases with an increasing number of iterations. When these bounds coincide, we have provably found an optimal solution, otherwise, we get near-optimal solutions with a quality guarantee. Assuming an upper bound on the number of interaction matches per line, which is typically the case with base pair probability matrices of RNA sequences, we get a running time of *O*(*n*^2^) for each Lagrangian iteration. Since we fix the number of iterations, this leads to an overall time complexity of *O*(*n*^2^).

For the multiple case, similar in spirit to the MARNA software, we combine our pairwise method with the popular progressive alignment software T-COFFEE [50]. Progressive methods build multiple alignments from pairwise alignments. The pairwise distances are usually used to compute a guide tree which in turn determines the order in which the sequences are aligned to the evolving multiple alignment.

Progressive approaches often suffer from their sensitivity to the order in which the sequences are chosen during the alignment process. T-COFFEE reduces this effect by making use of local alignment information from *all *pairwise sequence alignments during its progressive alignment phase. We supply such local alignment information based on all-against-all structural alignments computed with our pairwise approach, assigning a high score to conserved interaction matches. The structural information is subsequently passed on to T-COFFEE that computes a multiple alignment, taking into account the additional structural information.

## 3 Experiments

The basis of our computational experiments is the recently published benchmark set BRALIBASE 2.1 [51]. We compared our program to four other alignment programs (MARNA, FOLDALIGNM, MAFFT, and STRAL) using two established measures for the quality of structural alignments (Compalign and SCI score). We performed all experiments with default parameters.

### 3.1 BRAliBase 2.1

We chose this data set, which is available from [52], as our test set, since it covers a greater range of typical noncoding-RNA families than the original BRALIBASE data set [12]. BRALIBASE 2.1 contains 36 different RNA families, ranging from approximately 26 nucleotides long Histone 3'UTR stem-loop motifs to approximately 300 nucleotides long eukaryotic SRP RNAs. See [51] for a detailed listing of all instances. BRALIBASE 2.1 reference alignments are based on manually curated seed alignments of the *Rfam 7.0 *database [53]. Out of the pool of all ncRNA families that have more than 50 sequences in their seed alignment, either 2, 3, 5, 7, 10 or 15 sequences were randomly drawn considering constraints on the sequences (e.g., average pairwise sequence identity or structural conservation). These subsets of the original seed alignments form the instances of BRALIBASE: in the following we stick to the BRALIBASE naming convention and refer to the sets of instances by *k*2, *k*3, *k*5, *k*7, *k*10, and *k*15, depending on the number of sequences per instance.

### 3.2 Compalign and SCI

We use two different scores to measure the quality of the computed alignments: the *Compalign *value codes the degree of similarity to a given reference alignment as given by the percentage of columns that are identically aligned as in the reference alignment. A value of 1 states that the reference and test alignment are the same, whereas 0 denotes that no column was correctly aligned with respect to the reference alignment.

The second score is the so called *structural conservation index *[54] (or SCI in short). The SCI basically gives the degree of conservation of a consensus structure induced by a multiple alignment in relation to the *minimum free energy *structure of each sequence (to be more precise, not the actual structures are compared but their respective energy values). A SCI value of ≈ 1 indicates very high structural conservation, whereas a value around 0 indicates no structural conservation at all. Note that the SCI score can be greater than 1, because covariance information is additionally rewarded in the computation.

We have used the programs *compalignp *and *scif *to compute the Compalign and SCI score. Both tools are freely available from the BRAliBase website.

### 3.3 Other structural alignment programs

We implemented our approach called LARA in C++ within the LISA framework. LISA (*Li*brary of *S*tructural *A*lignment algorithms) contains various methods for aligning protein and RNA structures as well as biological networks.

Furthermore, we selected several other multiple structural alignment programs to compare the results. We used MARNA [26] (available from [55]) using an ensemble of three suboptimal structures as its input, STRAL [24] (a sequence based algorithm incorporating McCaskill's base pair probabilities, available from [56]), and a reimplementation of the PMCOMP approach called FOLDALIGNM [32] (a banded variant of Sankoff's algorithm that aligns base pair probability matrices, available from [57]). Furthermore, to compare the performance of the structure-based alignment programs to purely sequence-based ones, we performed the same tests with MAFFT [58], a recent multiple sequence alignment program which is available from [59]. We want to emphasize that we did not perform any parameter tuning for any program (this includes LARA), i.e., we downloaded the programs from the respective websites and performed the computations out of the box without specifying any optional parameters.

Since earlier studies [12,51] showed that structural alignments only contribute an additional benefit – compared to sequence-based approaches – if the pairwise sequence identity drops below ≈ 50 – 60%, we restricted the test set to instances of low homology, i.e., instances having a pairwise sequence identity below 50%.

### 3.4 LaRA

A scoring system for structural alignments has to provide two different kinds of scores: scores for the sequence and the structure part (in case of LARA, these correspond to weights for the alignment and interaction edges, respectively). Since the structure is considered to contain the necessary information for "correct" alignments, we have to make sure that the structure scores contribute the major part to the overall score.

We do not generate the complete annotation for our input sequence, that is, an interaction edge between every possible interaction, but restrict interaction edges to those having base pair probabilities larger than a threshold *p*_min_. For our experiments we resorted to a value of 0.003, similar as in PMCOMP. The impact of different *p*_min _values is twofold: First, the lower the value is, the higher the structure scores are. Secondly, a high *p*_min _value leads to a sparser structure graph.

For the scoring of the edges, LARA provides two different schemes: First, a scoring system based on base pair probability matrices (BPP scoring in short) that rescales the scores in spirit of PMCOMP. More precisely, given the probability *p*_*ij *_that nucleotide *i *and *j *pair, the actual score *s*_*ij *_for the structural interaction between *i *and *j *is given by

sij=lg⁡(pijpmin⁡)
 MathType@MTEF@5@5@+=feaafiart1ev1aaatCvAUfKttLearuWrP9MDH5MBPbIqV92AaeXatLxBI9gBaebbnrfifHhDYfgasaacH8akY=wiFfYdH8Gipec8Eeeu0xXdbba9frFj0=OqFfea0dXdd9vqai=hGuQ8kuc9pgc9s8qqaq=dirpe0xb9q8qiLsFr0=vr0=vr0dc8meaabaqaciaacaGaaeqabaqabeGadaaakeaacqWGZbWCdaWgaaWcbaGaemyAaKMaemOAaOgabeaakiabg2da9iGbcYgaSjabcEgaNnaabmaabaWaaSaaaeaacqWGWbaCdaWgaaWcbaGaemyAaKMaemOAaOgabeaaaOqaaiabdchaWnaaBaaaleaacyGGTbqBcqGGPbqAcqGGUbGBaeqaaaaaaOGaayjkaiaawMcaaaaa@4078@

where lg is the natural logarithm. For the sequence scoring, we take the entries from the RIBOSUM matrices [47] as the actual sequence scores (that is the scores for pairs of nucleotides) and multiply them by a user-specific adjustment factor *τ*. The default value for *τ *is 0.05, leading to a small sequence score contribution to the overall score. If one knows, however, that sequence is equally or more important than the structure (e.g., in case of riboswitches), one simply has to increase the value of *τ*.

The second scheme employs the RIBOSUM scoring matrices both for sequence and structure scoring: these matrices are based on given alignments of ribosomal RNAs from which log-odds scores were derived. They provide both sequence and structure scores, without rescaling the scores.

**Figure 12:**
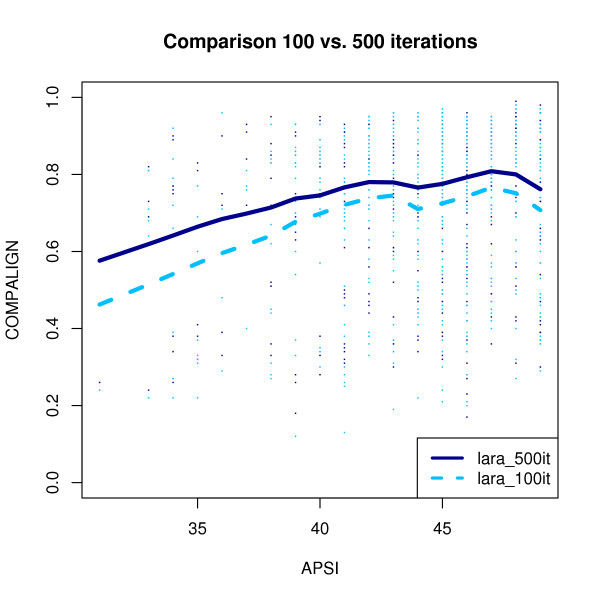
**Different number of iterations**. Comparison of all *k*10 instances of low homology between LARA running 100 or 500 iterations. Each dot correponds to one problem instance, the thick lines were computed using Lowess regression. The x-axis gives the average pairwise sequence identity (APSI). The y-axis codes the Compalign score.

The second crucial LARA parameter is the number of iterations: the more iterations LARA computes, the more often the penalty terms are adapted (yielding better alignments). As one can see in Fig. 12 the number of iterations influences the quality of the computed alignment while the running time increases linearly with the number of iterations. In our experiments we set the number of iterations to 500.

The scoring of gap edges follows the scheme of *affine gap costs *with an gap open and extension penalty of -6 and -2, respectively.

#### Score vs. alignment accuracy

**Figure 13:**
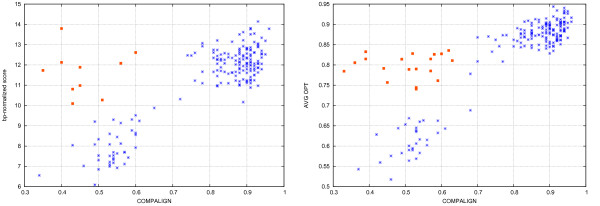
**Score vs. alignment accuracy**. All 189 BRALIBASE *k*10 instances of low pairwise sequence identity where each cross or box corresponds to one instance: The x-axis gives the Compalign score. The y-axis codes either a structure-normalized score (left side), or the optimality ratio (right side). The red boxes mark the outliers.

We were interested to what extent the accuracy of our alignments correlates with the actual BPP score that we computed. Since the score depends on the length of the input sequences, we normalized the score with respect to the number of paired bases in the *minimum free energy *structure. Note that we did not use the actual structure, but the number of base pairs in the structure to get a rough estimate of how many pairings we expect in the structure. Then, let p^
 MathType@MTEF@5@5@+=feaafiart1ev1aaatCvAUfKttLearuWrP9MDH5MBPbIqV92AaeXatLxBI9gBaebbnrfifHhDYfgasaacH8akY=wiFfYdH8Gipec8Eeeu0xXdbba9frFj0=OqFfea0dXdd9vqai=hGuQ8kuc9pgc9s8qqaq=dirpe0xb9q8qiLsFr0=vr0=vr0dc8meaabaqaciaacaGaaeqabaqabeGadaaakeaacuWGWbaCgaqcaaaa@2E25@ and *n *be the average score and the number of base pairs in the MFE structure, then the *base-pair normalized score *is given by p^
 MathType@MTEF@5@5@+=feaafiart1ev1aaatCvAUfKttLearuWrP9MDH5MBPbIqV92AaeXatLxBI9gBaebbnrfifHhDYfgasaacH8akY=wiFfYdH8Gipec8Eeeu0xXdbba9frFj0=OqFfea0dXdd9vqai=hGuQ8kuc9pgc9s8qqaq=dirpe0xb9q8qiLsFr0=vr0=vr0dc8meaabaqaciaacaGaaeqabaqabeGadaaakeaacuWGWbaCgaqcaaaa@2E25@/*n*. The left side of Fig. 13 shows the results for all 189 *k*10 instances with an average pairwise sequence identity less than 50%. The great majority of instances behaves as expected: the higher the bp-score is, the better is the corresponding Compalign score: There is, however, a group of 10 outliers (represented by the red boxes). Although they have a high bp-score (greater than 10.0), the alignment accuracy is bad: it turned out that these 10 instances are all SECIS-elements, indicating that the BPP scoring scheme is not appropriate for this group.

Furthermore, we assumed that there should a correlation between the actual performance of our algorithm and, again, the quality of our alignments: Remember that each Lagrange iteration results in a new valid solution and a new upper bound for the problem instance. Dividing the value of the highest lower bound by the value of the lowest upper bound gives an *optimality ratio*, i.e., a measure of how close the best solution is to an optimal one. Assuming an inverse correlation between the gap between lower and upper bound and the quality of the alignment, we again took all *k*10 BRALIBASE instances of low pairwise sequence identity and computed the arithmetic mean of the optimality ratios of all pairwise alignments. The right side of Fig. 13 shows the plot for all 189 *k*10 instances with a sequence similarity lower than 50%. Most of the instances behave as expected: the higher the average optimality ratio is, the closer is the computed alignment to the reference alignment (and vice versa). There is, however, a group of 19 instances that behave differently (marked as red boxes in Fig. 13): Although their average optimality ratio is high (*> *0.7), the corresponding Compalign value is rather low compared to instances of a similar average optimality ratio. A closer inspection revealed that all instances of the upper left corner (that is instances having a Compalign value lower than 0.65 and an average optimality ratio of greater than 0.7, represented by red boxes in Fig. 13) comprises almost all instances of either bacterial SRP RNAs or SECIS elements (just one SRP RNA instance is not among the 19 instances). We therefore increased the number of iterations for one SECIS instance to see whether this would positively influence the quality of the alignment. By setting the number of iterations to 500, 1000, and 2000 we got average optimality ratios of 0.83, 0.85, and 0.87, by simultaneously yielding Compalign values of 0.39, 0.38, and 0.36, respectively. Obviously, the better the computed alignments in terms of the optimality ratio are, the worse they got with respect to the reference alignment.

**Figure 14:**
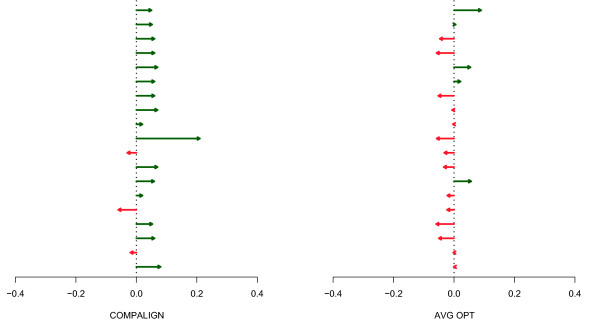
**Changing the scoring from BPP to RIBOSUM**. Change of the Compalign score and optimality ratio after changing the scoring from BPP to RIBOSUM matrices for the 19 outlier instances.

Consequently, for the outlier instances described above, we changed the scoring from BPP to RIBOSUM scores. Figure 14 shows the change in terms of the Compalign score and optimality ratio for the 19 outlier instances: 16 instances had better Compalign scores by using the RIBOSUM scoring, whereas the optimality ratio decreased in the majority of instances.

**Figure 15:**
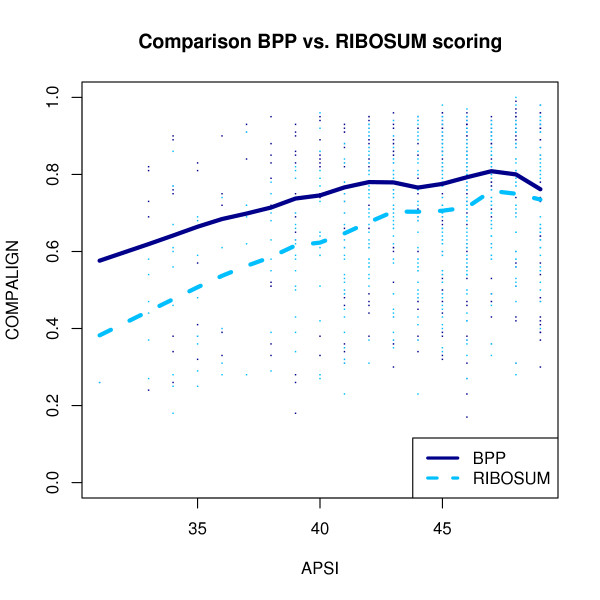
**BPP vs. RIBOSUM scoring**. Comparison between base pair probability (BPP) and RIBOSUM scoring. The x-axis gives the average pairwise sequence identity (APSI). The y-axis codes the Compalign score.

In general, however, our experiments showed that RIBOSUM scoring is not superior to BPP scoring (at least for the BRALIBASE benchmark and LARA): Figure 15 shows a comparison of all low homology *k*5 instances using either base pair probability matrices or RIBOSUM scoring, and it is obvious that base pair probability scoring yields better results on these input instances.

### 3.5 Comparison with other programs

As described in Sect. 3.2 we used two different scores to assess the quality of the computed alignments: the Compalign (the degree of similarity between the test alignment to a given reference alignment) and the SCI score (the degree of structural conservation induced by the test alignment).

FOLDALIGNM performs an alignment and clustering of the input sequences at the same time: in some instances, FOLDALIGNM splits the input sequences into two clusters. Since the scores that we use depend on the number of input sequences, we dropped those FOLDALIGNM alignments that did not contain all sequences in the final alignment: This leads to 43, 30, 11, 15, 19, and 6 instances that we did not consider in case of *k*2, *k*3, *k*5, *k*7, *k*10, and *k*15 instances.

**Figure 16:**
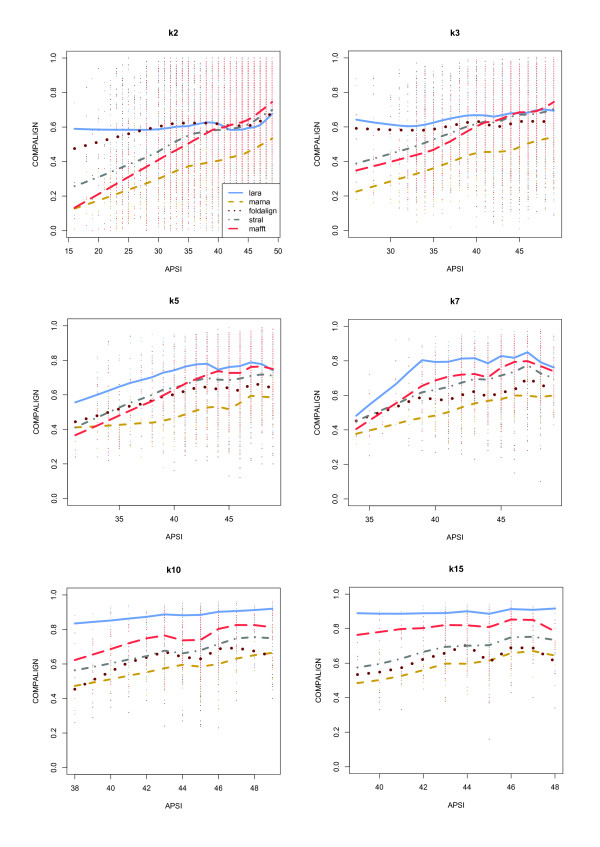
**Compalign results on low homology instances**. Results on all low homology instances containing 2 (upper left), 3 (upper right), 5 (middle left), 7 (middle right), 10 (lower left), and 15 (lower right) instances from the BRALIBASE benchmark set. The *x*- and *y*-axes give the average pairwise sequence identity (APSI) and the Compalign score. The legend from the upper left plot applies to the other plots as well. Mind that the different APSI-ranges in the six plots are a result of the BRALIBASE benchmark set: there are, for example, no *k*15 instances in BRALIBASE below 38%.

In Fig. 16 we show the results of our experiments broken down to the different input classes (either *k*2, *k*3, *k*5, *k*7, *k*10, or *k*15). These graphics have the average pairwise sequence identity and the Compalign score as their *x*- and *y*-axis, respectively. The reference alignments therefore correspond to horizontal lines at a Compalign score of 1.0.

We have made several observations: First of all, in the pairwise case (i.e., the *k*2 instances) LARA has a similar performance as the Sankoff variant FOLDALIGNM up to a sequence identity of ≈ 42%. For the range of ≈ 42 – 50% all programs (even sequence-based MAFFT) have comparable performance (except for MARNA). With an increasing number of input sequences per instance, especially for the *k*10 and *k*15 sequences, the results change tremendously: LARA outperforms the other programs, yielding average Compalign scores of ≈ 90%, whereas the other structure-based alignment programs have scores around ≈ 55 – 75%. This is quite remarkable, especially considering that FOLDALIGNM and LARA show a similar performance in the pairwise case: FOLDALIGNM, however, computes multiple alignments in a progressive fashion, whereas LARA computes *all *pairwise alignments and leaves it to T-COFFEE to compute an alignment that is highly consistent with all pairwise alignments. With an increasing number of input sequences, the consistency-based approach generates better alignments than the progressive methods (at least in the case of our experimental setup).

Another astonishing observation is the performance of MAFFT, a purely sequence-based program: the *k*2 and *k*3 instances show a comparable performance for instances above ≈ 42%, which is already surprising. With a growing number of input instances, the performance of MAFFT becomes even better: in case of 15 input instances, the program yields – on average – the second best results (behind LARA), outperforming even FOLDALIGNM and STRAL, which incorporate structural information. It has to be investigated whether the creation of the benchmark set has to be revisited, because these plots clearly contradict the hypothesis that sequence-based programs yields significantly worse results for input instances of a pairwise sequence identity below 50%.

**Figure 17:**
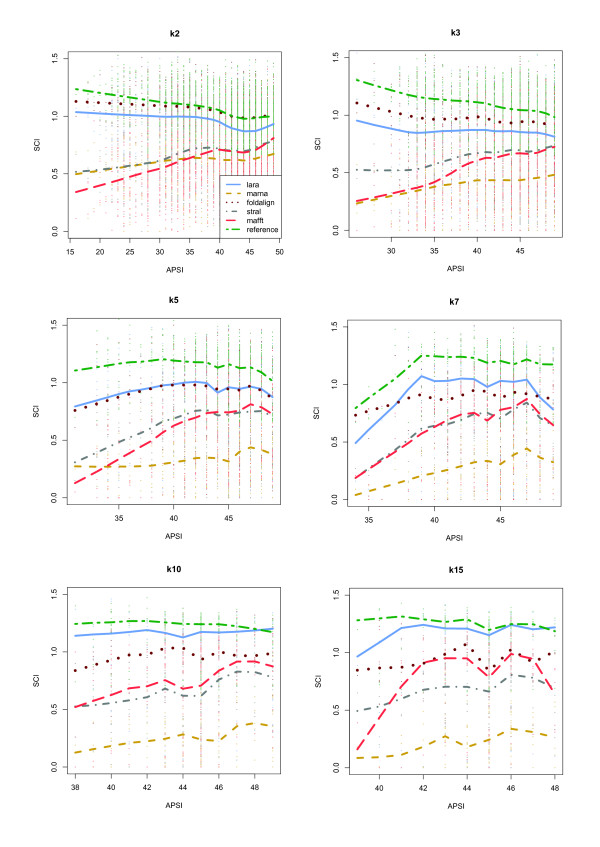
**SCI results on low homology instances**. Results on all low homology instances containing 2 (upper left), 3 (upper right), 5 (middle left), 7 (middle right), 10 (lower left), and 15 (lower right) instances from the BRALIBASE benchmark set. The *y*-axis gives the SCI score. The legend from the upper left plot applies to the other plots as well. Mind that the different APSI-ranges in the six plots are a result of the BRALIBASE benchmark set: there are, for example, no *k*15 instances in BRALIBASE below 38%.

In Fig. 17 we show the results with respect to the SCI score (remember that the SCI is a measure for the structural conservation of an alignment). The general trend is the same as in Fig. 16. In the pairwise case, the LARA curve has the same shape as the reference curve, but shifted to the bottom by about 0.1. FOLDALIGNM yields the best approximation to the reference line, having almost the same performance for instances with an APSI greater than 30%. With an increasing number of input sequences, the situation changes: from *k*5 on LARA generates the best approximation to the reference line, with FOLDALIGNM being the second best program. Taking a look at the various result plots puts the extraordinary performance of MAFFT into perspective regarding the *k*10 and *k*15 input sets.

#### Comparison of running times

We compared the programs tested on the same computing server with an Intel Xeon CPU running at 3.2 GHz, 3.5 GB RAM, and Linux kernel version 2.6.16. It turned out that memory requirement was not an issue, but the computation time instead: especially MARNA scales in O
 MathType@MTEF@5@5@+=feaafiart1ev1aaatCvAUfKttLearuWrP9MDH5MBPbIqV92AaeXatLxBI9gBaebbnrfifHhDYfgasaacH8akY=wiFfYdH8Gipec8Eeeu0xXdbba9frFj0=OqFfea0dXdd9vqai=hGuQ8kuc9pgc9s8qqaq=dirpe0xb9q8qiLsFr0=vr0=vr0dc8meaabaqaciaacaGaaeqabaqabeGadaaakeaat0uy0HwzTfgDPnwy1egaryqtHrhAL1wy0L2yHvdaiqaacqWFoe=taaa@383C@(*n*^4^), which makes the alignment of longer sequences (for example the SRP instances of BRALIBASE) rather time-consuming. This, however, is not the case with LARA and FOLDALIGNM, since these two programs have running times in O
 MathType@MTEF@5@5@+=feaafiart1ev1aaatCvAUfKttLearuWrP9MDH5MBPbIqV92AaeXatLxBI9gBaebbnrfifHhDYfgasaacH8akY=wiFfYdH8Gipec8Eeeu0xXdbba9frFj0=OqFfea0dXdd9vqai=hGuQ8kuc9pgc9s8qqaq=dirpe0xb9q8qiLsFr0=vr0=vr0dc8meaabaqaciaacaGaaeqabaqabeGadaaakeaat0uy0HwzTfgDPnwy1egaryqtHrhAL1wy0L2yHvdaiqaacqWFoe=taaa@383C@(*n*^2^). To evaluate the time consumption within reasonable time, we therefore set a time limit of 20 minutes per instance: If the computation was not finished within 20 minutes, the process was killed and we took 20 minutes as the actual running time. In Table 2 we list the number of instances that the corresponding program was not able to align within 20 minutes.

**Table 2:** Failed instances. Unsolved instances within a time limit of 20 minutes.

Program	*k*2	*k*3	*k*5	*k*7	*k*10	*k*15
LARA	0	0	0	0	0	0
FOLDALIGNM	0	0	0	0	0	0
STRAL	0	0	0	0	0	0
MARNA	0	49	23	17	12	6
MAFFT	0	0	0	0	0	0

**Figure 18:**
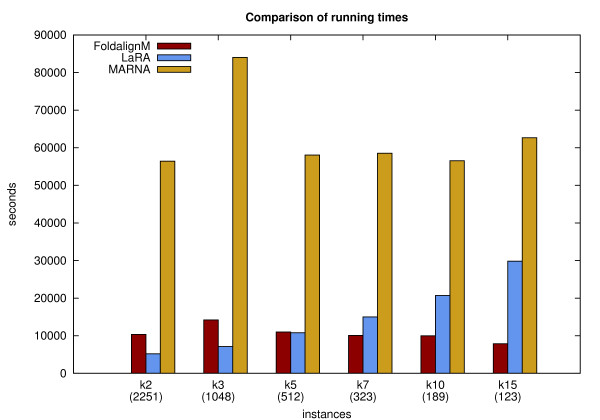
**Running times of sequence-structure alignment programs**. The plot shows a comparison of the running times between the structural programs tested. With an increasing number of input sequences, a progressive alignment strategy pays off compared to the computation of all pairwise alignments. The *x*-axis lists the different input instances, either containing 2, 3, 5, 7, 10, or 15 sequences (denoted by *k*2, *k*3, *k*5, *k*7, *k*10, and *k*15, respetively). The numbers in brackets denote the number of instances per input class. The y-axis gives the number gives the computation time in seconds.

We were especially interested in how the running times of the programs that use structure information scaled with respect to the number of the input sequences: FOLDALIGNM is a progressive approach which computes (*n *- 1) pairwise alignments given *n *input sequences. MARNA and LARA, however, compute all n(n−1)2
 MathType@MTEF@5@5@+=feaafiart1ev1aaatCvAUfKttLearuWrP9MDH5MBPbIqV92AaeXatLxBI9gBaebbnrfifHhDYfgasaacH8akY=wiFfYdH8Gipec8Eeeu0xXdbba9frFj0=OqFfea0dXdd9vqai=hGuQ8kuc9pgc9s8qqaq=dirpe0xb9q8qiLsFr0=vr0=vr0dc8meaabaqaciaacaGaaeqabaqabeGadaaakeaadaWcaaqaaiabd6gaUjabcIcaOiabd6gaUjabgkHiTiabigdaXiabcMcaPaqaaiabikdaYaaaaaa@3407@ pairwise alignments. Figure 18 shows the execution time of all five programs on all *k*2, *k*3, *k*5, *k*7, *k*10, and *k*15 instances. As one can see, with an increasing number of input sequences, a progressive alignment strategy pays off compared to the computation of all pairwise alignments.

## 4 Conclusion

We have presented a novel method for computing high-quality pairwise structural RNA alignments. We approach the original problem using a flexible graph-based model, which naturally deals with pseudoknots.

We find solutions in our model by means of an integer linear programming formulation and the Lagrangian relaxation technique. For the multiple case, we compute all-against-all pairwise solutions and pass this information to T-COFFEE, a progressive alignment algorithm.

Our extensive computational experiments on a large set of benchmark alignments show that LARA, the implementation of our algorithm, is competitive with state-of-the art tools and outperforms alternative approaches with an increasing number of input sequences. The difference to other programs gets larger the more sequences that have to be aligned. In this context, we also find the performance of MAFFT, a purely sequence-based program, remarkable. MAFFT comes closer to manually curated reference alignments than all other structure-specific tools besides LARA for alignments of more than ten sequences.

Our plans for the future include a local version of our alignment algorithm. Furthermore, we are currently implementing an exact branch-and-bound framework around the Lagrangian approach and will develop a stem-based variant of LARA. Furthermore, the openness to pseudoknots is the main advantage of LARA over alternative approaches, and we plan to adapt our method to produce high-quality alignments of pseudoknotted structures.

## Availability and requirements

LARA (Lagrangian relaxed alignments) is part of the **C++ **library LiSA and is freely available for academic purposes from . The binary runs under the Linux operating system.

All alignments that we computed and the scripts for generating the plots are also available from .

## Authors' contributions

MB, GWK, and KR developed the integer linear program model. MB coded the program and carried out the computational experiments. MB and GWK drafted the manuscript, GWK and KR coordinated the research. All authors read and approved the final manuscript.

## Supplementary Material

Additional file 1 This is a compressed tar file containing all alignments used in the experimental study. Click here for file
